# Investigations of In Vitro Anti-Acetylcholinesterase, Anti-Diabetic, Anti-Inflammatory, and Anti-Cancer Efficacy of Garden Cress (*Lepidium sativum* Linn.) Seed Extracts, as Well as In Vivo Biochemical and Hematological Assays

**DOI:** 10.3390/pharmaceutics17040446

**Published:** 2025-03-31

**Authors:** Ahmed M. Naglah, Abdulrahman A. Almehizia, Mohamed A. Al-Omar, Asma S. Al-Wasidi, Mayada H. Mohamed, Sanad M. Alsobeai, Ashraf S. Hassan, Wael M. Aboulthana

**Affiliations:** 1 Drug Exploration and Development Chair (DEDC), Department of Pharmaceutical Chemistry, College of Pharmacy, King Saud University, P.O. Box 2457, Riyadh 11451, Saudi Arabia; mehizia@ksu.edu.sa (A.A.A.); malomar1@ksu.edu.sa (M.A.A.-O.); 2 Department of Chemistry, College of Science, Princess Nourah Bint Abdulrahman University, Riyadh 11671, Saudi Arabia; asalwasidi@pnu.edu.sa; 3 University Family Medicine Center, Department of Family and Community Medicine, College of Medicine, King Saud University Medical City, P.O. Box 2925, Riyadh 11472, Saudi Arabia; mabdallah@ksu.edu.sa; 4 Department of Biological Sciences, College of Science and Humanities, Shaqra University, P.O. Box 1040, Ad-Dawadimi 11911, Saudi Arabia; salsobaei@su.edu.sa; 5 Organometallic and Organometalloid Chemistry Department, National Research Centre, Dokki, Cairo 12622, Egypt; 6 Biochemistry Department, Biotechnology Research Institute, National Research Centre, Dokki, Cairo 12622, Egypt; wmkamel83@hotmail.com

**Keywords:** garden cress (*Lepidium sativum* Linn.) seed, in vitro biological assays, Alzheimer’s disease, antioxidant activity, cytotoxic activity, toxicity

## Abstract

**Background/Objectives:** The current research was designed to quantify the active phyto-constituents and investigate the in vitro biological efficiency of different garden cress (*Lepidium sativum* Linn.) seed extracts against chronic diseases as well as the in vivo toxicities that may be induced in mice upon the administration of each extract at both studied therapeutic doses. **Methods**: The in vitro biological efficiency of different *L. sativum* extracts, such as methanolic, aqueous, acetone, and ethyl acetate extracts, was assessed. The inhibition percentage (%) and the median inhibitory concentration (IC_50_) values of different *L. sativum* extracts were estimated against acetylcholinesterase enzyme, diabetes mellitus (α-amylase and α-glucosidase enzymes), and inflammation (cyclooxygenase-1 (COX-1), cyclooxygenase-2 (COX-2), and 5-lipoxygenase (5-LOX) enzymes). Additionally, the median inhibitory concentration (IC_50_) values of different L. sativum extracts against HepG-2, Caco-2, and A549 cells were assessed using 3-(4,5-dimethythiazol-2-yl)-2,5-diphenyl tetrazolium bromide (MTT) assay. Moreover, the toxicities that might be induced in mice at hematological (using an automatic blood analyzer) and biochemical levels were evaluated. **Results:** It was found that the methanolic *L. sativum* extract possessed the highest in vitro biological activities compared to the other studied extracts. The inhibition percentage values of the methanolic extract were 51.34, 54.35, 44.10, 43.48, and 40.78% against acetylcholinesterase, α-amylase, α-glucosidase, protein denaturation, and proteinase enzymes, respectively. The methanolic extract also exhibited an inhibitory effect against the COX-1 (55.05%), COX-2 (57.30%), and 5-LOX (50.15%) enzymes. Additionally, the methanolic extract possesses the highest cytotoxic activity against HepG-2, Caco-2, and A549 cells, with IC_50_ values of 52.27, 40.73, and 37.95 μg/mL, respectively. The median lethal doses (LD_50_) showed that the methanolic extract was safer when administered orally, followed by aqueous and acetone, then ethyl acetate extract. It was found that methanolic, aqueous, and acetone extracts showed no alterations when administered orally at two studied doses (1/10 and 1/20 of LD_50_) compared to the control. **Conclusions**: This study concluded that the methanolic extract possessed the highest in vitro biological activities and was safer than the other studied extracts, followed by aqueous, acetone, and then ethyl acetate extract. In the future, the in vivo biological efficacy of the methanolic *L. sativum* extract will be evaluated, as well as an elucidation of its mechanism against chronic diseases.

## 1. Introduction

Alzheimer’s disease, inflammation, diabetes mellitus, and cancer belong to chronic diseases affecting the nervous system [1,2,3,4,5]. Free radicals cause oxidative stress, which plays an important role in cell death, tissue and DNA damage, cancer, aging, oxidized side chains of proteins, and inflammation. Thus, they disrupt receptors and neurotransmitter functions. Oxidative stress causes neurological disorders and neuroinflammation [6]. Therefore, it is important for humans to consume antioxidants for protection against various diseases and the protection of the nervous system [7].

Diabetes mellitus is an imbalance in blood glucose levels and is separated into type 1 and type 2 categories [8]. This poses risks to human health and various complications, such as diabetic retinopathy, Alzheimer’s disease, cardiovascular disease, and damage to the liver and kidneys. Also, diabetes mellitus can cause nerve damage, known as diabetic neuropathy, which affects the autonomic and peripheral nervous systems [9].

Alzheimer’s disease is a neurological disorder that causes brain cell damage, leading to a decrease in neurotransmitters such as acetylcholine, which results in memory problems [10]. The accumulation of amyloid β protein, neuritic plaques, neurofibrillary tangle formation, and increased tau levels causes Alzheimer’s disease [11]. Alzheimer’s disease causes abnormalities in brain tissue. As a result of these abnormalities, it has effects on the central nervous system and leads to the deterioration of the nerves that control the body’s functions [12].

Cancer is a disease characterized by uncontrolled cell growth and division [13]. There are types of cancer related to the nerves, such as pheochromocytoma, brain cancer, and neuroblastoma. Cancer cells can affect the nerves and cause nerve damage and inflammation [14]. The autonomic nervous system is affected by direct or indirect cancer-related complications [15].

A wide range of plants have been regarded as the primary source of potent therapeutic drugs for centuries [16]. Garden cress (*Lepidium sativum* Linn.) is one of these plants. *L. sativum* belongs to the Brassicaceae (Cruciferae) family and the genus Lepidium. It is categorized as a fast-growing edible herbaceous plant, and its seeds are reddish-brown in color [17,18]. Regarding the seeds of *L. sativum*, they are used in the therapy of diverse diseases, where they possess antioxidant, anti-inflammatory, anti-microbial properties, cardio-protective, hepato-protective, and nephroprotective effects [19]. Moreover, these seeds incorporate high levels of abundant phytochemicals, such as phenols, terpenoids, alkaloids, flavonoids, saponins, anthracene glycosides, carbohydrates, proteins, minerals, and fibers [20]. Getahun et al. demonstrated that 1-isocyano-2-methylbenzene was the predominant compound in the essential oils of *L. sativum* seeds and also proved the antioxidant and antibacterial activities of these oils [21].

In addition, the *L. sativum* aglycone extract mitigated liver microsomal lipid and protein oxidation due to the presence of a high concentration of polyphenols in addition to organosulfur compounds [22]. Also, *L. sativum* leaf extract exhibits anti-inflammatory activities by modulating connective tissue and inhibiting the proliferation of fibroblasts [23]. *L. sativum* ethanolic extract inhibited the fungal growth [24]. Desai et al. proposed that *L. sativum* ethanolic extract improves the antioxidative status of the liver and pancreas in mice through scavenging free radicals [25].

Based on scientific information about Alzheimer’s disease, inflammation, diabetes mellitus, and cancer; the extracts, biological potential, and bioactive compounds of *L. sativum* seed; as well as the continuation of our research targets [26,27,28,29,30,31,32]. Consequently, the present study focuses on revealing the in vitro biological efficiency of different *L. sativum* seed extracts against acetylcholinesterase enzyme (Alzheimer’s disease), diabetes mellitus (α-amylase and α-glucosidase enzymes), and inflammation (cyclooxygenase-1 (COX-1), cyclooxygenase-2 (COX-2), and 5-lipoxygenase (5-LOX) enzymes). Additionally, the in vitro biological efficiency against HepG-2, Caco-2, and A549 cells was assessed as an anti-cancer agent. As well as, the in vivo toxicities that might be induced in mice at hematological and biochemical levels were evaluated.

## 2. Materials and Methods

### 2.1. Collection of Seeds and Preparation of Extracts

*L. sativum* seeds were purchased from a local market (the Egyptian market), shade-dried, and then ground to a fine powder. The methanolic, aqueous, acetone, and ethyl acetate extracts were prepared using the methods reported [33,34]. The methods reported are detailed in the Supplementary File.

### 2.2. Phyto-Chemical Evaluation of L. sativum Seed Extracts

Concentrations of total phenolic content, total condensed tannins, and total flavonoid content were quantified in all prepared extracts using the methods suggested by Singleton and Rossi (1965) [35], Broadhurst and Jones (1978) [36], and Arvouet-Grand et al. (1994) [37], respectively. The methods suggested are detailed in the Supplementary File.

### 2.3. In Vitro Studies of L. sativum Seed Extracts

Antioxidant [38,39], scavenging [40,41,42], anti-Alzheimer [43], anti-diabetic [44,45], anti-arthritic [46,47,48], anti-inflammatory [49,50], cytotoxic [51], and enzymatic [52,53,54] activities were assessed according to the proposed methods detailed in the Supplementary File. All biological activities were assessed in triplicate for all studied extracts.

### 2.4. In Vivo Studies of L. sativum Seed Extracts

#### 2.4.1. Median Lethal Dose (LD_50_)

The LD_50_ of the different extracts was studied separately according to the proposed method [55].

#### 2.4.2. Experimental Design

The details of the experimental design are explained in the Supplementary File.

#### 2.4.3. Sample Collections

The details of the sample collections [56] are explained in the Supplementary File.

#### 2.4.4. Biochemical Assays

##### Hematological and Biochemical Measurements

The hematological and biochemical measurements [57] are detailed in the Supplementary File.

##### Biochemical Assays in Supernatants of Tissues Homogenates

Oxidative stress markers [58,59,60,61,62,63,64] and levels of inflammatory markers [65,66] were determined according to the proposed methods detailed in the Supplementary File.

### 2.5. Statistical Analysis

The details of the statistical analysis are explained in the Supplementary File.

## 3. Results

### 3.1. Phyto-Chemical Evaluation of L. sativum Seed Extracts

The levels of the most common active phyto-constituents (total phenolic content, total condensed tannins, and total flavonoid contents) were measured in different *L. sativum* seed extracts (methanolic, aqueous, acetone, and ethyl acetate) (Table 1). The results showed that the levels of these phyto-constituents were higher in the methanolic extract (173.27 mg gallic acid equivalent/100 g, 77.01 μg/mL and 44.01 mg quercetin equivalent/100 g, respectively) compared to the other studied extracts. This extract was followed by aqueous and acetone, then ethyl acetate extract. Therefore, the lowest concentrations of these phyto-constituents were noticed in the ethyl acetate extract (50.41 mg gallic acid equivalent/100 g, 22.40 μg/mL, and 12.80 mg quercetin equivalent/100 g, respectively).

### 3.2. In Vitro Studies on L. sativum Seed Extracts

#### 3.2.1. Antioxidant Activity

The antioxidant activities (total antioxidant capacity (TAC) and iron reducing power (IRP)) of these extracts were assessed and compared to the standard drug (Table 1). As a result, the methanolic extract exhibited higher TAC and IRP (303.23 mg gallic acid/g and 295.48 µg/mL, respectively) compared to the other studied extracts. Based on the concentrations of the phyto-constituents, the extract was followed by aqueous and acetone, then ethyl acetate extract. Therefore, the lowest TAC and IRP (88.21 mg gallic acid/g and 80.46 µg/mL, respectively) were noticed in the ethyl acetate extract, whereas ascorbic acid was used as a standard showed lower antioxidant activity (84.50 mg gallic acid/g and 76.75 µg/mL, respectively) compared to all studied extracts.

#### 3.2.2. Scavenging Activity

The scavenging activities against various free radicals (namely, 1,1-diphenyl-2-picryl-hydrazyl (DPPH), 2,2′-azinobis-(3-ethylbenzothiazoline-6-sulfonic acid) (ABTS), and nitric oxide (NO)) of these extracts were assessed and compared to the standard drug (Table 2). Regarding the scavenging activities, the data showed that the methanolic extract exhibited higher scavenging activity (53.31%, 57.06%, and 47.56%, respectively) compared to the other studied extracts at the same concentration (100 µg/mL). Based on the data on antioxidant activity, the extract was followed by aqueous, acetone, and then ethyl acetate extract. Therefore, the lowest scavenging activity (15.51%, 19.26%, and 9.76%, respectively) was noticed in the ethyl acetate extract, whereas the ascorbic acid used as a standard at the same concentration showed the highest scavenging activity (66.50%, 70.25%, and 60.75%, respectively).

The IC_50_ values of DPPH, ABTS, and NO scavenging activities were calculated and are listed in Table 2. The extract with higher scavenging activity was observed to have lower IC_50_ values. Therefore, the lowest IC_50_ values were observed with the methanolic extract (5.32 µg/mL, 4.68 µg/mL, and 7.03 µg/mL, respectively) compared to the other extracts. Ascorbic acid exhibited the lowest IC_50_ values (4.29 µg/mL, 3.80 µg/mL, and 5.51 µg/mL, respectively) compared to all studied extracts.

#### 3.2.3. Anti-Alzheimer’s and Anti-Diabetic Activities

The anti-Alzheimer’s activity (acetylcholinesterase (AChE) enzyme) of these extracts was assessed and compared to the standard drug (Table 3). The data showed that the methanolic extract exhibited a higher inhibitory effect on the AChE enzyme (51.34%) compared to the other studied extracts at the same concentration. This extract was followed by the aqueous (41.07%), acetone (23.88%) extracts, and then the ethyl acetate extract (14.94%). Meanwhile, donepezil used as a standard at the same concentration showed the highest anti-Alzheimer’s activity (65.15%). Regarding the IC_50_ values, the lowest IC_50_ value was observed with the methanolic extract (5.35 µg/mL) compared to the other extracts. Donepezil exhibited the lowest IC_50_ values (4.21 µg/mL) compared to all studied extracts.

The anti-diabetic activity (α-amylase and α-glucosidase enzymes) of these extracts was assessed and compared to the standard drug (Table 3). It was found that the methanolic extract showed a higher inhibitory effect against the activities of both enzymes (54.35% and 44.10%, respectively) compared to the other studied extracts at the same concentration. This extract was followed by the aqueous and acetone extracts, and then the ethyl acetate extract. Meanwhile, acarbose used as a standard at the same concentration showed the highest anti-diabetic activity (66.90% and 56.65%, respectively). Regarding the IC_50_ values, the lowest IC_50_ value was observed with the methanolic extract (4.24 and 2.87 µg/mL, respectively) compared to the other extracts. Acarbose exhibited the lowest IC_50_ values (3.45 and 2.23 µg/mL, respectively) compared to all studied extracts.

#### 3.2.4. Anti-Arthritic and Anti-Inflammatory Activities

The anti-arthritic activity (protein denaturation and proteinase enzymes) of these extracts was assessed. The data depicted in Table 4 showed that the methanolic extract exhibited the highest anti-arthritic activity (43.48% and 40.78%, respectively) compared to the other studied extracts at the same concentration. This was followed by the aqueous and acetone extracts, and then the ethyl acetate extract. Therefore, the lowest anti-arthritic activity (14.67% and 11.97%, respectively) was observed in the ethyl acetate extract. Meanwhile, diclofenac sodium used as a standard at the same concentration showed the highest anti-arthritic activity (53.52% and 50.82%, respectively). The IC_50_ values showed that the lowest IC_50_ value was observed with the methanolic extract against the activity of the proteinase enzyme (7.84 µg/mL) compared to the other extracts. Diclofenac sodium exhibited the lowest IC_50_ values (6.29 µg/mL) compared to all the studied extracts.

The anti-inflammatory activities of these extracts against the cyclooxygenase-1 (COX-1), cyclooxygenase-2 (COX-2), and 5-lipoxygenase (5-LOX) enzymes were assessed. The data depicted in Table 4 showed that the methanolic extract exhibited the highest activity (55.05%, 57.30%, and 50.15%, respectively) compared to the other studied extracts at the same concentration. This was followed by the aqueous and acetone extracts, and then the ethyl acetate extract. Therefore, the lowest activity (16.17%, 18.42%, and 11.27%, respectively) was observed in the ethyl acetate extract. Meanwhile, indomethacin used as a standard at the same concentration showed the highest inhibitory activity against the activities of COX-1 and COX-2 enzymes (68.61% and 70.86%, respectively). In terms of the activity against the 5-LOX enzyme, zileuton was used as a standard and showed the highest inhibitory effect against the activity of this enzyme (55.71%). The IC_50_ values (Table 4) showed that the lowest IC_50_ values were observed with the methanolic extract against the activities of these enzymes (7.13 µg/mL, 5.27 µg/mL, and 8.01 µg/mL, respectively) compared to the other extracts. Indomethacin (5.72 µg/mL and 4.26 µg/mL) and zileuton (7.21 µg/mL) showed the lowest IC_50_ values compared to all the studied extracts.

Finally, the statistical analysis of all the results from the in vitro biological tests evaluated at equal concentrations was performed and showed a highly positive correlation with each other at *p* ≤ 0.01, according to the statistical correlations displayed in Table S1.

#### 3.2.5. Cytotoxic Activity and Enzymatic Assays

**Figure 1:**
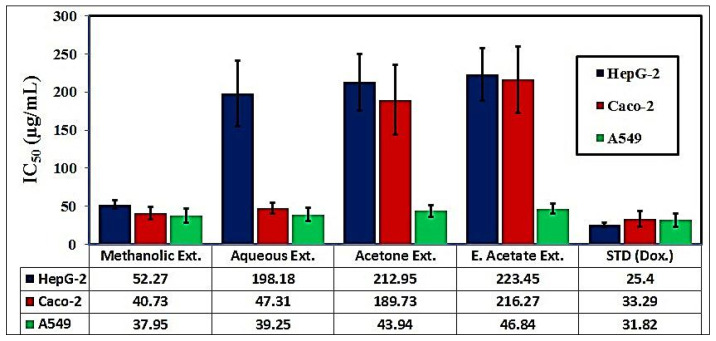
The in vitro cytotoxic activities of the different *L. sativum* seed extracts against HepG-2, Caco-2, and A549 cancer cell lines.

The in vitro cytotoxic activities of these extracts against human hepatocellular carcinoma (HepG-2), colon (Caco-2), and lung (A549) cancer cell lines were assessed and compared to the standard drug (Figure 1). The data showed that the methanolic extract exhibited the highest cytotoxic activity against HepG-2, Caco-2, and A549 cells with the lowest IC_50_ value (IC_50_ = 52.27, 40.73, and 37.95 μg/mL, respectively). This was followed by the aqueous extract (IC_50_ = 198.18, 47.31, and 39.25 μg/mL, respectively) and acetone extract (IC_50_ = 212.95, 189.73, and 43.94 μg/mL, respectively), and then the ethyl acetate extract (IC_50_ = 223.45, 216.27, and 46.84 μg/mL, respectively). Doxorubicin, which was used as a standard, showed the highest cytotoxic activity (IC_50_ = 25.40, 33.29, and 31.82 μg/mL, respectively) compared to all the studied extracts.

After performing the cytotoxicity assay, the safety of each extract was assessed by calculating the median lethal dose (LD_50_) following oral administration.

The efficiency of the different studied extracts against the activities of caspase-3 and Bcl-2 enzymes was evaluated in the three studied cancer cell lines (HepG-2, Caco-2, and A549) (Table 5). It was observed that the methanolic extract increased the activity of caspase-3 (310.05, 270.21, and 252.72 pg/mL, respectively) while decreasing the Bcl-2 level (4.95, 2.55, and 5.59 ng/mL, respectively) in the treated HepG-2, Caco-2, and A549 cells compared to the untreated cells. The highest activity of the caspase-3 enzyme (418.84, 365.02, and 341.39 pg/mL, respectively) and the lowest activity of the Bcl-2 enzyme (2.90, 1.49, and 3.27 pg/mL, respectively) were observed in HepG-2, Caco-2, and A549 cells treated with doxorubicin compared to all the studied extracts.

### 3.3. In Vivo Study on L. sativum Seed Extracts

#### 3.3.1. Median Lethal Dose (LD_50_)

**Figure 2:**
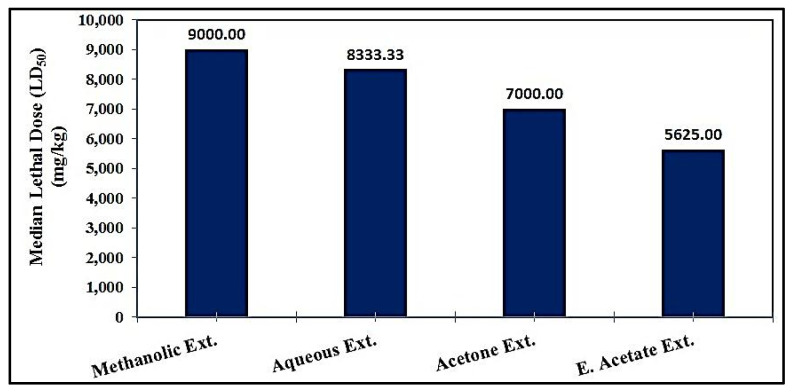
The median lethal doses (LD_50_) of different extracts studied from *L. sativum* seed in mice.

The therapeutic doses were determined as 1/10 and 1/20 of the median lethal dose (LD_50_). The data compiled in Table S2 show that the LD_50_ of the methanolic extract was calculated from a series of extract concentrations (1000, 2000, 4000, 6000, 8000, 10,000, 12,000, and 14,000 mg/kg). It was found that the LD_50_ was about 9000 mg/kg, and, therefore, the therapeutic doses (1/10 and 1/20 of LD_50_) were about 900 and 450 mg/kg, respectively. The LD_50_ of the aqueous extract was calculated from a series of extract concentrations (1000, 2000, 4000, 6000, 8000, 10,000, 12,000, and 14,000 mg/kg). It was found that the LD_50_ was about 8333.33 mg/kg. Therefore, the therapeutic doses were about 833.33 and 416.67 mg/kg, respectively (Table S3). The LD_50_ of the acetone extract was calculated from a series of extract concentrations (500, 1000, 2000, 4000, 6000, 8000, 10,000, and 12,000 mg/kg). It was found that the LD_50_ was about 7000 mg/kg. Therefore, the therapeutic doses were about 700 and 350 mg/kg, respectively (Table S4). Regarding the ethyl acetate extract, a series of extract concentrations (500, 1000, 2000, 4000, 6000, 8000, and 10,000 mg/kg) was used to calculate the LD_50_, which was about 5625 mg/kg. Therefore, the therapeutic doses were about 562.5 and 281.25 mg/kg, respectively (Table S5). The data presented in Figure 2 show that the methanolic extract had the highest LD_50_ value (9000 mg/kg), followed by the aqueous extract (8333.3 mg/kg) and acetone extract (7000 mg/kg), and then the ethyl acetate extract (5625 mg/kg).

#### 3.3.2. Hematological and Biochemical Measurements

Hematological measurements, including RBCs, HB, HCT, platelets, and WBC count, remained unchanged in all mice treated with the methanolic, aqueous, and acetone extracts at both studied doses (1/10 and 1/20 LD_50_) compared to the control group. Regarding the ethyl acetate extract, all hematological measurements decreased significantly (*p* ≤ 0.05) in the mice treated with 562.5 mg/kg compared to the control group. The group treated with 281.25 mg/kg showed a significant (*p* ≤ 0.05) decline in these measurements, but they were significantly (*p* ≤ 0.05) higher than in the group treated with 562.5 mg/kg (Table 6).

The biochemical measurements, including liver, kidney, and heart functions, as well as the lipid profile, remained unchanged in all mice treated with the methanolic, aqueous, and acetone extracts at both studied doses compared to the control group. In relation to the ethyl acetate extract, all biochemical measurements were adversely affected by a significant (*p* ≤ 0.05) increase in liver enzymes (ALT, AST, ALP, and GGT), kidney markers (urea, creatinine, and BUN), and heart indicators (CK and LDH), as well as lipid levels (TC, TGs, and LDL-c) in the mice treated with 562.5 mg/kg compared to the control group. The group treated with 281.25 mg/kg also showed a significant (*p* ≤ 0.05) increase in these measurements, but they were significantly (*p* ≤ 0.05) lower than those in the group treated with 562.5 mg/kg (Table 7).

#### 3.3.3. Biochemical Assays in Supernatants of Tissue Homogenates

In the liver and spleen tissues of the mice treated with methanolic, aqueous, and acetone extracts at both studied doses, the concentrations of enzymatic and non-enzymatic antioxidants remained unchanged. Regarding the ethyl acetate extract, all antioxidant measurements decreased significantly (*p* ≤ 0.05) in the mice treated with 562.5 mg/kg compared to the control group. The group treated with 281.25 mg/kg showed a significant (*p* ≤ 0.05) decline in these measurements, but they were significantly (*p* ≤ 0.05) higher than in the group treated with 562.5 mg/kg. In the kidney tissue, the concentrations of antioxidants remained unchanged upon treatment of the mice with all studied extracts at both studied doses (Table 8).

**Figure 3:**
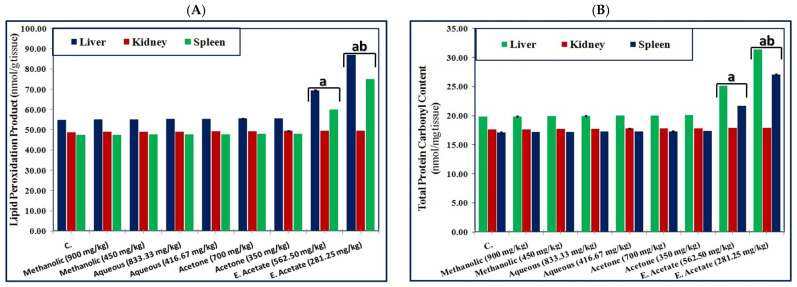
Effect of the different *L. sativum* seed extracts at two doses (1/10 and 1/20 of LD_50_) on (**A**) Concentration of the lipid peroxidation product and (**B**) total protein carbonyl contents in the most target organs of mice. Data were represented as mean ± SE, a: indicates a statistically significant difference compared to the control group, b: indicates a statistically significant difference compared to the same extract at the higher dose (1/10 of LD_50_) at *p* ≤ 0.05.

As for the products of the peroxidation reactions (LPO and TPC), the levels of these products remained unchanged in the mice treated with methanolic, aqueous, and acetone extracts at both studied doses. They increased significantly (*p* ≤ 0.05) in both liver and spleen tissues in the mice treated with 562.5 mg/kg compared to the control group. The group treated with 281.25 mg/kg showed a significant (*p* ≤ 0.05) increase in these measurements, but they were significantly (*p* ≤ 0.05) lower than in the group treated with 562.5 mg/kg. In the kidney tissue, the concentrations of antioxidants remained unchanged upon treatment of the mice with all studied extracts at both studied doses (Figure 3).

In the liver and spleen tissues of the mice treated with methanolic, aqueous, and acetone extracts at both studied doses, the levels of the inflammatory markers (TNF-α, IL-6) in addition to the activity of AChE remained unchanged. Regarding the ethyl acetate extract, these measurements increased significantly (*p* ≤ 0.05) in the mice treated with 562.5 mg/kg compared to the control group. The group treated with 281.25 mg/kg showed a significant (*p* ≤ 0.05) elevation in these measurements, but they were significantly (*p* ≤ 0.05) lower than in the group treated with 562.5 mg/kg. In the kidney tissue, the concentrations of antioxidants remained unchanged upon treatment of the mice with all studied extracts at both studied doses (Table 9).

## 4. Discussion

It is well known that plants have the ability to produce natural phyto-constituents with antioxidant properties that enable them to reduce the amount of oxidative stress caused by oxygen and sunlight [67]. *L. sativum* is characterized by its significant nutritional and therapeutic properties. Therefore, researchers from different regions of the world have investigated the nutritional profiling of the leaves and seeds of *L. sativum* in recent years [68].

During the current study, it was found that the methanolic extract is rich in high concentrations of active phyto-constituents (total phenolic content, total condensed tannins, and total flavonoids) (Table 1). This finding is supported by Chatoui et al., who reported that methanolic seed extract contains a significant phenolic and flavonoid content [69]. Kumar et al. reported that the phenolic and flavonoid content represented 0.5% and 0.375% in the methanolic seed extract, respectively [70].

Kassem et al. (2020) [71] demonstrated that the *L. sativum* methanolic extract contained five flavonoids, namely, kaempferol, quercetin, kaempferol-3-O-α-L-rhamnopyranoside, kaempferol-3-O-β-D-glucopyranoside, and quercetin-3-O-β-D-galactopyranoside. The five flavonoids were characterized using spectroscopic techniques. Additionally, Baregama et al. (2022) [72] proved that the methanolic extract contained 27 phytochemical compounds, some of which are as follows: 1-isocyano-3-methylbenzene (83.02%), (isothiocyanatomethyl)benzene or benzyl mustard oil (1.91%), 4-[(*S*)-1-methylpropyl]-2,3-dihydrofuran (4.02%), and 2,5-dimethylfuran (3.11%) (using GC-MS analysis).

The primary approach to treating chronic diseases induced by excessive lipid oxidation and inflammation, such as atherosclerosis, cancer, and rheumatoid arthritis, involves consuming exogenous antioxidants [73]. The methanolic extract exhibited higher TAC and IRP compared to the other studied extracts (Table 1). This was in accordance with Solaiman, who stated that there is a positive correlation between a high amount of phenolic compounds and the highest antioxidant ability [74]. This finding aligns with our results. Attia et al., supported that the *L. sativum* methanol extract was the most effective in the reducing power test [75]. Regarding the scavenging activity, it was found that the methanolic extract exhibited higher scavenging activity against DPPH, ABTS, and NO radicals compared to the other studied extracts at the same concentration (Table 2). This finding is consistent with Ouattar et al., who suggested that the antioxidant activity of a plant extract depends on the structure of bioactive compounds, such as phenolic acids and flavonoids, and their ability to neutralize reactive oxygen and nitrogen species. Therefore, the methanolic extract showed effective scavenging of free radicals and was considered a powerful inhibitor of free radicals due to the high yield of phenolic compounds [76].

Neurodegenerative diseases, among which one of the more common is Alzheimer’s disease, are characterized by the deterioration of cognitive function and memory, with progressive degeneration of the structure and function of the central or peripheral nervous system, resulting in cognitive decline and death [77]. AChE is a serine hydrolase enzyme primarily responsible for terminating the transmission of the nerve impulse at cholinergic synapses through the hydrolysis of the neurotransmitter (acetylcholine) into two inactive compounds (choline and acetic acid) [78]. The strategy for treating Alzheimer’s disease involves controlling the level of acetylcholine, a neurotransmitter in cholinergic synapses, by blocking the degradation of acetylcholine using AChE inhibitors. Therefore, efficient treatments for neurodegenerative diseases may involve the discovery of effective AChE activity inhibitors [79]. In the present study, it was noticed that the methanolic extract exhibited a higher inhibitory effect on the AChE enzyme compared to the other studied extracts at the same concentration (Table 3). This finding is consistent with El-Shamarka et al., who demonstrated that antioxidant and scavenging activities are strongly related to anti-Alzheimer’s activity [80]. Therefore, an extract that possesses antioxidant activities also exhibits anti-Alzheimer’s activity. Furthermore, the presence of the aglycone moiety structure and heterocyclic nitrogen of steroidal alkaloids plays an important feature in AChE inhibition [81]. Phenolic compounds interact with amino acid residues that define the active site of AChE through hydrogen bonding and hydrophobic interactions. The multiple hydroxyl groups in phenolic compounds enhance the inhibitory action of AChE due to their stronger binding capacity [82].

The characteristic that distinguishes diabetes mellitus (DM), a chronic metabolic disorder, is elevated glucose levels [83]. Carbohydrates are broken down by α-amylase into disaccharides and then by α-glucosidase into monosaccharides. It is believed that the most effective treatment is to reduce the activity of these enzymes to control hyperglycemia [84]. The anti-diabetic activity was assessed by measuring the inhibitory effect against the activity of the α-amylase enzyme and comparing it to the efficiency of acarbose, a standard drug [85]. During the present study, it was found that the methanolic extract possessed a high inhibitory effect on the activities of α-amylase and α-glucosidase enzymes compared to the other studied extracts at the same concentration (Table 3). This finding is consistent with Aboulthana et al., who demonstrated that the presence of phenolic acids and tannins is responsible for the inhibitory effect on the activities of these enzymes [86]. Furthermore, the amount and orientation of functional groups in the phenolic compounds, as well as the structure–activity relationship, may all be connected to how these enzymes are inhibited [87].

An autoimmune disease called arthritis is characterized by inflammation [88]. Both protein denaturation and the proteinase enzyme are responsible for inflammatory disorders such as arthritis. Therefore, it is believed that one potential therapy strategy for arthritis is to block them [89]. During the current study, the methanolic extract exhibited the highest inhibitory effect on both protein denaturation and the proteinase enzyme compared to the other studied extracts at the same concentration (Table 4). This might be attributable to increasing the scavenging activity against free radicals, which are key contributors to inflammation and arthritis [90]. Inhibition of the proteinase enzyme may involve hydrogen bonding between the hydroxyl groups of phenolics and oxygen or nitrogen, specifically hydroxyl and amino groups of proteins [91]. Additionally, the inhibition of the proteinase enzyme could be attributed to the associations between phenolic compounds with non-polar aromatic rings and hydrophobic regions of the protein molecules, which are the main reasons for hydrophobic interactions [92], and/or due to the electrostatic interactions between the hydroxyl groups of phenolics and charged groups on the proteins [93].

The 5-lipoxygenase (5-LOX) and cyclooxygenase (COX) are well-known pro-inflammatory enzymes that are frequently used to evaluate anti-inflammatory agents. The two COX isoforms, COX-1 and COX-2, are required for the metabolism of arachidonic acid to prostaglandins [94]. They differ greatly from each other in their chemical structures, intracellular locations, and biological roles, although they do not differ functionally [95]. During the current study, it was found that the methanolic extract exhibited the highest anti-inflammatory activity compared to the other studied extracts at the same concentration (Table 4). This might refer to increasing the concentration of the polyphenolic compounds, which have the ability to inhibit these enzymes [96]. Also, this might be attributed to capabilities of the flavonoids, which are influenced by a number of OH groups, conjugations, and resonances [97].

Cancer is a group of diseases characterized by malignant neoplasms arising from uncontrolled cell proliferation that invades and destroys the surrounding tissue, leading to death if not controlled [98]. During the present study, it was noticed that the methanolic extract exhibited the highest cytotoxic activity against HepG-2, Caco-2, and A549 cells with the lowest IC_50_ values (Figure 1). This is consistent with Cruz et al., who demonstrated that cytotoxic activity is related to the chemical composition of the extracts [99]. The extract with the highest cytotoxic activity towards cancer cells might be due to the presence of high concentrations of phenolic compounds, which reduced the viability of cancer cells, as observed in the MTT assay [100]. Huang et al. added that these phenolic and flavonoidal compounds exert their anti-proliferative activity by interacting with cell cycle regulatory effects, thereby reducing the proliferation process [101].

As proposed by Naglah et al., the extract with cytotoxic activity increased the activity of caspase-3 while decreasing the Bcl-2 level in the treated cancer cells compared to the untreated ones [84]. During the present study, the highest activity of the caspase-3 enzyme and the lowest activity of the Bcl-2 enzyme were observed in HepG-2, Caco-2, and A549 cells treated with doxorubicin compared to all the studied extracts (Table 5). This may be attributed to the highest concentrations of bioactive compounds, which have been reported to activate the release of caspase-3 for proapoptosis and inhibit the progression of the tumor cell cycle at the S-phase [102]. They also suppress the action of cyclin-dependent kinases (Cdk), thereby limiting the progression of the tumor cell cycle at the G1 phase [103]. Moreover, saponins and triterpenes stimulate apoptosis of cancer cells through the upregulation of caspase-3 and downregulation of Bax (proapoptotic protein) and Bcl-2 (antiapoptotic protein) secretion, leading to the disintegration of poly (ADP-ribose) polymerase accompanied by DNA fragmentation and condensation of nuclear chromatin [104,105].

Acute oral toxicity is usually an initial screening step in the evaluation of the toxic characteristics of the studied extracts [106]. The toxicity induced by the extract might be directly related to the doses it is exposed to. The extract might be toxic if given in large enough doses because the difference between the therapeutic purpose and the toxicity is the dose, which increases the toxicity with an increase in dose [107]. The data from the current study showed that the methanolic extract had the highest LD_50_ value, making it safer than the other studied extracts (Figure 2). This agrees with Luka et al., who postulated that the crude methanolic extract has low toxicity and is safe when administered orally in mice [108].

Hematological measurements play a crucial role in assessing the physiological and pathological status. Thus, alterations in the normal values of these measurements could occur following the ingestion of some toxic plants [109]. In the present study, it was observed that no significant alterations exist in all these measurements of the extract-treated mice when compared with the control group, except for the group treated with the ethyl acetate extract, which decreased these measurements in a dose-dependent manner (Table 6). This might be attributable to the hematopoiesis pathway, which was influenced by the extract without a cellular inflammatory process [110].

Activities of enzymes in body fluids and tissue enzymes are frequently used as “markers” to establish the toxic effects of the extracts administered to experimental animals [111]. The present study showed that the administration of the ethyl acetate extract elevated levels of biochemical measurements related to the integrity of liver, kidney, and heart tissues (Table 7). This may be related to the production of reactive oxygen species (ROS) and a reduction in endogenous antioxidant enzymes (SOD, GSH, and CAT), which are reliable markers of antioxidant status in tissues, as supported by the present study (Table 8). Consequently, this leads to an elevation in LPO and TPC (Figure 3), which are sensitive and reliable indicators of lipid peroxidation and protein oxidation [112]. Therefore, levels of the biochemical measurements were significantly elevated upon administration of the ethyl acetate extract compared to the control.

TNF-α and IL-6 are categorized as markers of the inflammatory response. They are considered central regulators of inflammation and immune imbalance [113]. The present study demonstrated that the administration of ethyl acetate extract increased the levels of the inflammatory markers (TNF-α, IL-6) and activity of the AChE enzyme in the liver and spleen tissues of the mice (Table 9). This finding might refer to the inflammatory conditions related to the production of excessive ROS and depletion of the antioxidant enzymes [114]. Furthermore, the expression of inflammatory mediators was upregulated by oxidative and nitrosative stress and triggered through the activation of the transcription factor nuclear factor kappa-B [115].

In the end, our study showed that the methanolic extract possesses the highest biological activity both in vitro and in vivo because it contains a variety of potent phyto-constituents that are well known for their biological efficacy [69,70,71,72,76]. Numerous polar compounds can be effectively extracted using methanol, a polar solvent with high solubility. Beyond phenolic chemicals, lipids, and fatty acids, methanol is also utilized to extract proteins, amino acids, lignans, terpenoids, anthocyanins, and polysaccharides [116]. Acetone is a highly volatile medium-polar solvent that works well for extracting medium-polar compounds, particularly flavonoids and phenolic compounds [117]. As a polar solvent, water is the safest and greenest extraction technique. It can be extracted without the need for specialized equipment and is reasonably priced. It is mostly used to extract water-soluble components, such as phenolic compounds, anthocyanins, lipids, fatty acids, polysaccharides, saponins, vitamins, and minerals, though the extraction efficiency may be low [118].

## 5. Conclusions

In this current study, we found that the methanolic *L. sativum* extract possessed the highest in vitro biological activities compared to the other studied extracts. This extract exhibited a highly inhibitory effect against AChE (51.34%), α-amylase (54.35%), α-glucosidase (44.10%), protein denaturation (43.48%), and proteinase enzyme (40.78%). It also showed a highly inhibitory effect against COX-1 (55.05%), COX-2 (57.30%), and 5-LOX (50.15%). Furthermore, the methanolic extract exhibited the lowest IC_50_ values against HepG-2 (52.27 μg/mL), Caco-2 (40.73 μg/mL), and A549 (37.95 μg/mL) cells compared to the other studied extracts at the same concentration.

In the in vivo study assessing the toxicity induced in mice by the administration of different extracts at two doses (1/10 and 1/20 of LD_50_), it was observed that methanolic, aqueous, and acetone extracts did not cause any hematological or biochemical alterations (in liver and spleen tissues) compared to the control group. But the ethyl acetate extract caused alterations in a dose-dependent manner.

Finally, the methanolic *L. sativum* extract was found to be biologically promising. Thus, we recommend that advanced studies of this extract be performed in the future to evaluate its biological activities and elucidate its mechanism against Alzheimer’s disease (anti-AChE enzyme), inflammation (anti-COX-1, anti-COX-2, and anti-5-LOX), diabetes mellitus (anti-α-amylase and anti- α-glucosidase), and cancer diseases in rats.

## Figures and Tables

**Table 1:** Major phyto-constituents and in vitro antioxidant activity of different *L. sativum* seed extracts.

Extracts	Major Phyto-Constituents			Antioxidant Activities	
	Total Phenolic Content (mg Gallic Acid Equivalent/100 g)	Total Condensed Tannins (μg/mL)	Total Flavonoid (mg Quercetin Equivalent/100 g)	TAC (mg Gallic Acid/g)	IRP (µg/mL)
**Methanolic**	173.27 ± 1.19	77.01 ± 0.53	44.01 ± 0.30	303.23 ± 2.08	295.48 ± 2.08
**Aqueous**	138.62 ± 0.95	61.61 ± 0.42	35.20 ± 0.24	242.58 ± 1.66	234.83 ± 1.66
**Acetone**	80.59 ± 0.55	35.82 ± 0.25	20.47 ± 0.14	141.04 ± 0.97	133.29 ± 0.97
**Ethyl Acetate**	50.41 ± 0.34	22.40 ± 0.15	12.80 ± 0.09	88.21 ± 0.60	80.46 ± 0.60
**STD** **(Ascorbic Acid)**	-	-	-	84.50 ± 0.13	76.75 ± 0.13

**Table 2:** Inhibition percentage (%) and the median inhibitory concentrations (IC_50_, µg/mL) of the scavenging activities (DPPH, ABTS, and NO) of the different *L. sativum* seed extracts.

Extracts	Inhibition (%)			IC_50_ (µg/mL)		
	DPPH	ABTS	NO	DPPH	ABTS	NO
**Methanolic**	53.31 ± 0.36	57.06 ± 0.36	47.56 ± 0.36	5.32 ± 0.02	4.68 ± 0.03	7.03 ± 0.04
**Aqueous**	42.65 ± 0.29	46.40 ± 0.29	36.90 ± 0.29	6.69 ± 0.06	5.75 ± 0.03	9.07 ± 0.05
**Acetone**	24.80 ± 0.17	28.55 ± 0.17	19.05 ± 0.17	11.54 ± 0.04	9.42 ± 0.06	17.56± 0.12
**Ethyl Acetate**	15.51 ± 0.11	19.26 ± 0.11	9.76 ± 0.11	18.25 ± 0.07	13.88 ± 0.06	34.28 ± 0.30
**STD** **(Ascorbic Acid)**	66.50 ± 0.11	70.25 ± 0.11	60.75 ± 0.11	4.29 ± 0.03	3.80 ± 0.04	5.51 ± 0.02

The inhibition percentages were calculated at a concentration of 100 µg/mL for each of the tested extracts.

**Table 3:** Inhibition percentage (%) and the median inhibitory concentrations (IC_50_, µg/mL) of the anti-Alzheimer’s and anti-diabetic activities of the different *L. sativum* seed extracts.

Extracts	Anti-Alzheimer Activity		Anti-Diabetic Activity			
	AChE		α-Amylase		α-Glucosidase	
	Inhibition (%)	IC_50_ (µg/mL	Inhibition (%)	IC_50_ (µg/mL	Inhibition (%)	IC_50_ (µg/mL)
**Methanolic**	51.34 ± 0.35	5.35 ± 0.04	54.35 ± 0.35	4.24 ± 0.03	44.10 ± 0.35	2.87 ± 0.05
**Aqueous**	41.07 ± 0.28	6.68 ± 0.05	44.19 ± 0.28	5.22 ± 0.04	33.94 ± 0.28	3.73 ± 0.06
**Acetone**	23.88 ± 0.16	11.50 ± 0.09	27.19 ± 0.16	8.48 ± 0.06	16.94 ± 0.16	7.47 ± 0.14
**Ethyl Acetate**	14.94 ± 0.10	18.38 ± 0.14	18.34 ± 0.10	12.57 ± 0.09	8.09 ± 0.10	15.64 ± 0.33
**STD**	**Donepezil**		**Acarbose**			
	65.15 ± 0.12	4.21 ± 0.02	66.90 ± 0.10	3.45 ± 0.03	56.65 ± 0.10	2.23 ± 0.02

The inhibition percentages were calculated at a concentration of 100 µg/mL for each of the tested extracts.

**Table 4:** Inhibition percentage (%) and the median inhibitory concentrations IC_50_ (µg/mL) of the in vitro anti-arthritic and anti-inflammatory activities of the different *L. sativum* seed extracts.

Activities			STD	Methanolic	Aqueous	Acetone	Ethyl Acetate
**Anti-arthritic** **activity**	**Protein Denaturation (%)**		53.52 ± 0.08 (Diclofenac Sodium)	43.48 ± 0.28	35.35 ± 0.22	21.75 ± 0.13	14.67 ± 0.08
	**Proteinase**	**(%)**	50.82 ± 0.08 (Diclofenac Sodium)	40.78 ± 0.28	32.65 ± 0.22	19.05 ± 0.13	11.97 ± 0.08
		**IC_50_ (µg/mL)**	6.29 ± 0.02 (Diclofenac Sodium)	7.84 ± 0.04	9.79 ± 0.05	16.77 ± 0.08	26.68 ± 0.13
**Anti-inflammatory** **activity**	**COX-1**	**(%)**	68.61 ± 0.11 (Indomethacin)	55.05 ± 0.38	44.08 ± 0.30	25.72 ± 0.17	16.17 ± 0.11
		**IC_50_ (µg/mL)**	5.72 ± 0.02 (Indomethacin)	7.13 ± 0.08	8.90 ± 0.10	15.26 ± 0.18	24.28 ± 0.28
	**COX-2**	**(%)**	70.86 ± 0.11 (Indomethacin)	57.30 ± 0.38	46.33 ± 0.30	27.97 ± 0.17	18.42 ± 0.11
		**IC_50_ (µg/mL)**	4.26 ± 0.03 (Indomethacin)	5.27 ± 0.04	6.52 ± 0.05	10.79 ± 0.08	16.39 ± 0.11
	**5-LOX**	**(%)**	55.71 ± 0.11 (Zileuton)	50.15 ± 0.38	39.18 ± 0.30	20.82 ± 0.17	11.27 ± 0.11
		**IC_50_ (µg/mL)**	7.21 ± 0.03 (Zileuton)	8.01 ± 0.07	10.26 ± 0.09	19.31 ± 0.18	27.35 ± 0.05

The inhibition percentages were calculated at a concentration of 100 µg/mL for each of the tested extracts.

**Table 5:** Enzymatic assay values after treating cancer cells (HepG-2, Caco2, and A549) with different *L. sativum* seed extracts.

Extracts	The Median Inhibitory Concentrations (IC_50_)					
	HepG-2		Caco-2		A549	
	Caspase-3 (pg/mL)	Bcl-2 (ng/mL)	Caspase-3 (pg/mL)	Bcl-2 (ng/mL)	Caspase-3 (pg/mL)	Bcl-2 (ng/mL)
**DMSO**	108.79 ± 0.85	11.15 ± 0.15	94.81 ± 0.14	5.74 ± 0.08	88.67 ± 0.12	12.58 ± 0.05
**Methanolic**	310.05 ± 2.42	4.95 ± 0.07	270.21 ± 0.41	2.55 ± 0.04	252.72 ± 0.34	5.59 ± 0.02
**Aqueous**	212.14 ± 1.66	6.76 ± 0.09	222.80 ± 0.34	3.28 ± 0.05	208.38 ± 0.28	7.19 ± 0.03
**Acetone**	201.26 ± 1.57	8.92 ± 0.12	175.40 ± 0.26	4.59 ± 0.06	164.05 ± 0.22	10.06 ± 0.04
**Ethyl Acetate**	157.75 ± 1.23	10.62 ± 0.14	137.47 ± 0.21	5.47 ± 0.08	128.58 ± 0.17	11.98 ± 0.04
**STD (Doxorubicin** **)**	418.84 ± 3.27	2.90 ± 0.04	365.02 ± 0.55	1.49 ± 0.02	341.39 ± 0.46	3.27 ± 0.01

**Table 6:** Effect of different *L. sativum* seed extracts at two doses (1/10 and 1/20 of LD_50_) on hematological measurements in mice.

		C.	Methanolic		Aqueous		Acetone		Ethyl Acetate	
			900 (mg/kg)	450 (mg/kg)	833.33 (mg/kg)	416.67 (mg/kg)	700 (mg/kg)	350 (mg/kg)	562.50 (mg/kg)	281.25 (mg/kg)
**Formed Elements**	**RBCs (10^6^/µL)**	7.38 ± 0.02	7.39 ± 0.02	7.37 ± 0.02	7.38 ± 0.02	7.40 ± 0.02	7.41 ± 0.02	7.43 ± 0.02	5.62 ± 0.02 ^a^	6.46 ± 0.02 ^ab^
	**HB (g/dL)**	16.24 ± 0.06	16.27 ± 0.06	16.22 ± 0.06	16.25 ± 0.06	16.29 ± 0.06	16.32 ± 0.06	16.35 ± 0.06	12.36 ± 0.05 ^a^	14.22 ± 0.06 ^ab^
	**HCT (%)**	45.07 ± 0.05	45.16 ± 0.05	45.02 ± 0.05	45.11 ± 0.05	45.20 ± 0.05	45.28 ± 0.05	45.37 ± 0.05	34.31 ± 0.04 ^a^	39.46 ± 0.04 ^ab^
	**PLT (10^3^/µL)**	489.27 ± 1.60	490.24 ± 1.60	488.68 ± 1.60	489.64 ± 1.60	490.60 ± 1.60	491.57 ± 1.61	492.54 ± 1.61	372.43 ± 1.22 ^a^	428.30 ± 1.40 ^ab^
	**WBCs (10^3^/µL)**	9.38 ± 0.01	9.39 ± 0.01	9.36 ± 0.01	9.38 ± 0.01	9.40 ± 0.01	9.42 ± 0.01	9.44 ± 0.01	7.14 ± 0.01 ^a^	8.21 ± 0.01 ^ab^
**Differential Count**	**Lymp. (10^3^/µL)**	8.13 ± 0.01	8.15 ± 0.01	8.12 ± 0.01	8.14 ± 0.01	8.15 ± 0.01	8.17 ± 0.01	8.18 ± 0.01	6.19 ± 0.01 ^a^	7.12 ± 0.01 ^ab^
	**Mono. (10^3^/µL)**	0.74 ± 0.01	0.74 ± 0.01	0.74 ± 0.01	0.74 ± 0.01	0.74 ± 0.01	0.74 ± 0.01	0.74 ± 0.01	0.56 ± 0.01 ^a^	0.65 ± 0.01 ^ab^
	**Gran. (10^3^/µL)**	0.51 ± 0.01	0.51 ± 0.01	0.50 ± 0.01	0.51 ± 0.01	0.51 ± 0.01	0.51 ± 0.01	0.51 ± 0.01	0.38 ± 0.01 ^a^	0.44 ± 0.01 ^ab^

Data were represented as mean ± SE, ^a^: indicates a statistically significant difference compared to the control group; ^b^: indicates a statistically significant difference compared to the same extract at the higher dose (1/10 of LD_50_) at *p* ≤ 0.05.

**Table 7:** Effect of different *L. sativum* seed extracts at two doses (1/10 and 1/20 of LD_50_) on biochemical measurements in mice.

		C.	Methanolic		Aqueous		Acetone		Ethyl Acetate	
			900 (mg/kg)	450 (mg/kg)	833.33 (mg/kg)	416.67 (mg/kg)	700 (mg/kg)	350 (mg/kg)	562.50 (mg/kg)	281.25 (mg/kg)
**Liver**	**ALT (U/L)**	43.55 ± 0.01	43.64 ± 0.01	43.50 ± 0.01	43.58 ± 0.01	43.67 ± 0.01	43.76 ± 0.01	43.84 ± 0.01	63.02 ± 0.02 ^a^	54.80 ± 0.01 ^ab^
	**AST (U/L)**	63.16 ± 0.01	63.28 ± 0.01	63.08 ± 0.01	63.20 ± 0.01	63.33 ± 0.01	63.45 ± 0.01	63.58 ± 0.01	91.39 ± 0.01 ^a^	79.47 ± 0.01 ^ab^
	**ALP (U/L)**	97.23 ± 0.01	97.42 ± 0.01	97.11 ± 0.01	97.30 ± 0.01	97.50 ± 0.01	97.69 ± 0.01	97.88 ± 0.01	140.70 ± 0.02 ^a^	122.35 ± 0.01 ^ab^
	**GGT (U/L)**	24.51 ± 0.01	24.56 ± 0.01	24.48 ± 0.01	24.53 ± 0.01	24.58 ± 0.01	24.63 ± 0.01	24.67 ± 0.01	35.47 ± 0.01 ^a^	30.84 ± 0.01 ^ab^
**Kidney**	**Urea (mg/dL)**	38.45 ± 0.02	38.52 ± 0.02	38.40 ± 0.02	38.48 ± 0.02	38.55 ± 0.02	38.63 ± 0.02	38.70 ± 0.02	55.64 ± 0.03 ^a^	48.38 ± 0.02 ^ab^
	**Creat. (mg/dL)**	1.26 ± 0.01	1.26 ± 0.01	1.26 ± 0.01	1.26 ± 0.01	1.26 ± 0.01	1.27 ± 0.01	1.27 ± 0.01	1.82 ± 0.01 ^a^	1.59 ± 0.01 ^ab^
	**BUN (mg/dL)**	8.79 ± 0.03	8.81 ± 0.03	8.78 ± 0.03	8.80 ± 0.03	8.82 ± 0.03	8.84 ± 0.03	8.85 ± 0.03	12.73 ± 0.04 ^a^	11.07 ± 0.03 ^ab^
	**T. Protein (g/dL)**	8.30 ± 0.01	8.32 ± 0.01	8.29 ± 0.01	8.31 ± 0.01	8.32 ± 0.01	8.34 ± 0.01	8.36 ± 0.01	5.35 ± 0.01 ^a^	6.69 ± 0.01 ^ab^
	**Albumin (g/dL)**	3.96 ± 0.01	3.96 ± 0.01	3.95 ± 0.01	3.96 ± 0.01	3.97 ± 0.01	3.98 ± 0.01	3.98 ± 0.01	2.55 ± 0.01 ^a^	3.19 ± 0.01 ^ab^
**Heart**	**CK (U/L)**	70.56 ± 0.03	70.70 ± 0.03	70.48 ± 0.03	70.62 ± 0.03	70.76 ± 0.03	70.90 ± 0.03	71.03 ± 0.03	102.11 ± 0.04 ^a^	88.79 ± 0.03 ^ab^
	**LDH (U/L)**	225.12 ± 0.08	225.56 ± 0.08	224.84 ± 0.08	225.29 ± 0.08	225.73 ± 0.08	226.17 ± 0.08	226.62 ± 0.08	325.77 ± 0.12 ^a^	283.27 ± 0.10 ^ab^
**Lipid**	**TC (mg/dL)**	89.81 ± 0.02	89.99 ± 0.02	89.70 ± 0.02	89.88 ± 0.02	90.05 ± 0.02	90.23 ± 0.02	90.41 ± 0.02	129.96 ± 0.03 ^a^	113.01 ± 0.02 ^ab^
	**T.Gs (mg/dL)**	89.82 ± 0.01	90.00 ± 0.01	89.71 ± 0.01	89.89 ± 0.01	90.06 ± 0.01	90.24 ± 0.01	90.42 ± 0.01	129.98 ± 0.02 ^a^	113.02 ± 0.02 ^ab^
	**HDL-c (mg/dL)**	18.75 ± 0.02	18.79 ± 0.02	18.73 ± 0.02	18.77 ± 0.02	18.80 ± 0.02	18.84 ± 0.02	18.88 ± 0.02	9.14 ± 0.01 ^a^	13.60 ± 0.02 ^ab^
	**LDL-c (mg/dL)**	53.09 ± 0.02	53.20 ± 0.02	53.03 ± 0.02	53.13 ± 0.02	53.24 ± 0.02	53.34 ± 0.02	53.45 ± 0.02	76.83 ± 0.03 ^a^	66.81 ± 0.03 ^ab^

Data were represented as mean ± SE, ^a^: indicates a statistically significant difference compared to the control group; ^b^: indicates a statistically significant difference compared to the same extract at the higher dose (1/10 of LD_50_) at *p* ≤ 0.05.

**Table 8:** Effect of different *L. sativum* seed extracts at two doses (1/10 and 1/20 of LD_50_) on measurements of the antioxidant system in the most targeted organs of mice.

		C.	Methanolic		Aqueous		Acetone		Ethyl Acetate	
			900 (mg/kg)	450 (mg/kg)	833.33 (mg/kg)	416.67 (mg/kg)	700 (mg/kg)	350 (mg/kg)	562.50 (mg/kg)	281.25 (mg/kg)
**Liver**	**TAC (µmol/g)**	10.35 ± 0.01	10.37 ± 0.01	10.39 ± 0.01	10.41 ± 0.01	10.43 ± 0.01	10.45 ± 0.01	10.47 ± 0.01	6.70 ± 0.01 ^a^	8.38 ± 0.01 ^ab^
	**GSH (mg/g tissue)**	144.51 ± 0.06	144.79 ± 0.06	145.08 ± 0.06	145.37 ± 0.06	145.65 ± 0.06	145.94 ± 0.06	146.23 ± 0.06	93.58 ± 0.04 ^a^	116.98 ± 0.05 ^ab^
	**SOD (IU/g tissue)**	54.95 ± 0.01	55.06 ± 0.01	55.17 ± 0.01	55.28 ± 0.01	55.39 ± 0.01	55.50 ± 0.01	55.61 ± 0.01	35.59 ± 0.01 ^a^	44.49 ± 0.01 ^ab^
	**CAT (IU/g tissue)**	94.35 ± 0.02	94.54 ± 0.02	94.73 ± 0.02	94.91 ± 0.02	95.10 ± 0.02	95.29 ± 0.02	95.47 ± 0.02	61.10 ± 0.01 ^a^	76.38 ± 0.02 ^ab^
	**GPx (IU/g tissue)**	73.84 ± 0.02	73.98 ± 0.02	74.13 ± 0.02	74.28 ± 0.02	74.42 ± 0.02	74.57 ± 0.02	74.72 ± 0.02	47.82 ± 0.01 ^a^	59.77 ± 0.02 ^ab^
**Kidney**	**TAC (µmol/g)**	9.20 ± 0.01	9.22 ± 0.01	9.24 ± 0.01	9.26 ± 0.01	9.28 ± 0.01	9.30 ± 0.01	9.31 ± 0.01	9.35 ± 0.01	9.33 ± 0.01
	**GSH (mg/g tissue)**	128.30 ± 0.06	128.55 ± 0.06	128.81 ± 0.06	129.06 ± 0.06	129.31 ± 0.06	129.57 ± 0.06	129.82 ± 0.06	130.34 ± 0.06	130.08 ± 0.06
	**SOD (IU/g tissue)**	48.80 ± 0.01	48.90 ± 0.01	48.99 ± 0.01	49.09 ± 0.01	49.19 ± 0.01	49.29 ± 0.01	49.38 ± 0.01	49.58 ± 0.01	49.48 ± 0.01
	**CAT (IU/g tissue)**	83.78 ± 0.02	83.94 ± 0.02	84.11 ± 0.02	84.27 ± 0.02	84.44 ± 0.02	84.61 ± 0.02	84.77 ± 0.02	85.11 ± 0.02	84.94 ± 0.02
	**GPx (IU/g tissue)**	65.57 ± 0.02	65.69 ± 0.02	65.82 ± 0.02	65.95 ± 0.02	66.08 ± 0.02	66.21 ± 0.02	66.34 ± 0.02	66.61 ± 0.02	66.48 ± 0.02
**Spleen**	**TAC (µmol/g)**	8.95 ± 0.01	8.97 ± 0.01	8.99 ± 0.01	9.01 ± 0.01	9.03 ± 0.01	9.04 ± 0.01	9.06 ± 0.01	5.80 ± 0.01 ^a^	7.25 ± 0.01 ^ab^
	**GSH (mg/g tissue)**	124.76 ± 0.05	125.01 ± 0.05	125.25 ± 0.05	125.50 ± 0.05	125.75 ± 0.06	125.99 ± 0.06	126.24 ± 0.06	80.80 ± 0.04 ^a^	100.99 ± 0.04 ^ab^
	**SOD (IU/g tissue)**	47.46 ± 0.01	47.55 ± 0.01	47.65 ± 0.01	47.74 ± 0.01	47.83 ± 0.01	47.93 ± 0.01	48.02 ± 0.01	30.73 ± 0.01 ^a^	38.42 ± 0.01 ^ab^
	**CAT (IU/g tissue)**	81.47 ± 0.02	81.63 ± 0.02	81.79 ± 0.02	81.95 ± 0.02	82.11 ± 0.02	82.27 ± 0.02	82.44 ± 0.02	52.76 ± 0.01 ^a^	65.95 ± 0.01 ^ab^
	**GPx (IU/g tissue)**	63.76 ± 0.02	63.89 ± 0.02	64.01 ± 0.02	64.14 ± 0.02	64.26 ± 0.02	64.39 ± 0.02	64.52 ± 0.02	41.29 ± 0.01 ^a^	51.61 ± 0.01 ^ab^

Data were represented as mean ± SE, ^a^: indicates a statistically significant difference compared to the control group; ^b^: indicates a statistically significant difference compared to the same extract at the higher dose (1/10 of LD_50_) at *p* ≤ 0.05.

**Table 9:** Effect of different *L. sativum* seed extracts at two doses (1/10 and 1/20 of LD_50_) on markers of inflammatory reactions in the most targeted organs of mice.

		C.	Methanolic		Aqueous		Acetone		Ethyl Acetate	
			900 (mg/kg)	450 (mg/kg)	833.33 (mg/kg)	416.67 (mg/kg)	700 (mg/kg)	350 (mg/kg)	562.50 (mg/kg)	281.25 (mg/kg)
**Liver**	**TNF-α** **(pg/g tissue)**	215.24 ± 0.72	215.66 ± 0.73	216.09 ± 0.73	216.51 ± 0.73	216.94 ± 0.73	217.37 ± 0.73	217.80 ± 0.73	340.31 ± 1.14 ^a^	272.24 ± 0.92 ^ab^
	**IL-6** **(pg/g tissue)**	256.50 ± 0.51	257.01 ± 0.51	257.51 ± 0.51	258.02 ± 0.51	258.53 ± 0.51	259.04 ± 0.51	259.55 ± 0.51	405.54 ± 0.80 ^a^	324.43 ± 0.64 ^ab^
	**AChE** **(ng/g tissue)**	1.27 ± 0.01	1.27 ± 0.01	1.27 ± 0.01	1.27 ± 0.01	1.28 ± 0.01	1.28 ± 0.01	1.28 ± 0.01	2.00 ± 0.01 ^a^	1.60 ± 0.01 ^ab^
**Kidney**	**TNF-α** **(pg/g tissue)**	190.04 ± 0.28	190.41 ± 0.28	190.79 ± 0.28	191.16 ± 0.28	191.54 ± 0.28	191.92 ± 0.28	192.30 ± 0.29	193.05 ± 0.29	192.67 ± 0.29
	**IL-6** **(pg/g tissue)**	228.90 ± 0.45	229.35 ± 0.45	229.81 ± 0.45	230.26 ± 0.45	230.71 ± 0.46	231.17 ± 0.46	231.62 ± 0.46	232.54 ± 0.46	232.08 ± 0.46
	**AChE** **(ng/g tissue)**	0.89 ± 0.01	0.89 ± 0.01	0.89 ± 0.01	0.89 ± 0.01	0.90 ± 0.01	0.90 ± 0.01	0.90 ± 0.01	0.90 ± 0.01	0.90 ± 0.01
**Spleen**	**TNF-α** **(pg/g tissue)**	83.78 ± 0.47	83.95 ± 0.47	84.11 ± 0.47	84.28 ± 0.47	84.44 ± 0.47	84.61 ± 0.47	84.78 ± 0.47	132.46 ± 0.74 ^a^	105.97 ± 0.59 ^ab^
	**IL-6** **(pg/g tissue)**	99.08 ± 0.17	99.27 ± 0.17	99.47 ± 0.17	99.66 ± 0.18	99.86 ± 0.18	100.06 ± 0.18	100.25 ± 0.18	156.65 ± 0.28 ^a^	125.32 ± 0.22 ^ab^
	**AChE** **(ng/g tissue)**	6.25 ± 0.01	6.26 ± 0.01	6.27 ± 0.01	6.29 ± 0.01	6.30 ± 0.01	6.31 ± 0.01	6.32 ± 0.01	9.88 ± 0.01 ^a^	7.90 ± 0.01 ^ab^

Data were represented as mean ± SE, ^a^: indicates a statistically significant difference compared to the control group; ^b^: indicates a statistically significant difference compared to the same extract at the higher dose (1/10 of LD_50_) at *p* ≤ 0.05.

## Data Availability

Data are contained within the article or Supplementary File.

## Supplementary Materials

The following supporting information can be downloaded at: https://www.mdpi.com/article/10.3390/pharmaceutics17040446/s1, Table S1: Statistical correlations among the different in vitro biological activities of various *L. sativum* extracts at equal concentrations (100 µg/mL); Table S2: Data used for calculating the median lethal dose (LD_50_) of methanolic *L. sativum* seed extract in mice; Table S3: Data used for calculating the median lethal dose (LD_50_) of aqueous *L. sativum* seed extract in mice; Table S4: Data used for calculating the median lethal dose (LD_50_) of acetone *L. sativum* seed extract in mice; Table S5: Data used for calculating the median lethal dose (LD_50_) of ethyl acetate *L. sativum* seed extract in mice; biological methods, and statistical analysis.

## Abbreviations

AChE	Acetylcholinesterase enzyme
ALP	Alkaline phosphatase
AST	Aspartate transaminase
ABTS	2,2′-Azinobis-(3-ethylbenzothiazoline-6-sulfonic acid)
BUN	Blood urea nitrogen
CAT	Catalase
CK	Creatin kinase
COX-1	Cyclooxygenase-1 enzyme
COX-2	Cyclooxygenase-2 enzyme
MTT	3-(4,5-Dimethythiazol-2-yl)-2,5-diphenyl tetrazolium bromide
DPPH	1,1-Diphenyl-2-picryl-hydrazyl
GGT	Gamma-glutamyl transferase
GPx	Glutathione peroxidase enzyme
HCT	Hematocrit
HB	Hemoglobin
HDL-c	High-density lipoprotein-cholesterol
Caco-2	Human colon cancer
HepG-2	Human hepatocellular carcinoma
A549	Human lung cancer
IL-6	Interleukin-6
IRP	Iron-reducing power
LDH	Lactate dehydrogenase
*L. sativum*	*Lepidium sativum* Linn.
LPO	Lipid peroxidation product
5-LOX	5-Lipoxygenase enzyme
LDL-c	Low-density lipoprotein-cholesterol
NO	Nitric oxide
PLT	Platelet count
RBCs	Red blood cells
GSH	Reduced glutathione
ALT	Serum alanine transaminase
SOD	Superoxide dismutase
IC_50_	The median inhibitory concentration
LD_50_	The median lethal doses
TAC	Total antioxidant capacity
TAC	Total antioxidant capacity
TC	Total cholesterol
TPC	Total protein carbonyl
TG	Triglycerides
TNF-α	Tumor necrosis factor-α
WBCs	White blood cells
