# Supplementary material for: Investigations of In Vitro Anti-Acetylcholinesterase, Anti-Diabetic, Anti-Inflammatory, and Anti-Cancer Efficacy of Garden Cress (Lepidium sativum Linn.) Seed Extracts, as Well as In Vivo Biochemical and Hematological Assays
Source: Pharmaceutics. 2025 Mar 31;17(4):446. doi: 10.3390/pharmaceutics17040446 (PMC12030678; doi:10.3390/pharmaceutics17040446)
Supplement: Supplementary file 1 [file pharmaceutics-17-00446-s001.zip › pharmaceutics-3512644-supplementary.pdf]

Supplementary File

For

Investigations of In Vitro Anti-Acetylcholinesterase, Anti-Diabetic, Anti-Inflammatory, and Anti-Cancer Efficacy of Garden Cress (*Lepidium sativum Linn.*) Seed Extracts, as Well as In Vivo Biochemical and Hematological Assays

Ahmed M. Naglah [1,*], Abdulrahman A. Almehizia [1], Mohamed A. Al-Omar [1], Asma S. Al-Wasidi [2], Mayada H. Mohamed [3], Sanad M. Alsobeai [4], Ashraf S. Hassan [5,*] and Wael M. Aboulthana [6]

[1] Drug Exploration and Development Chair (DEDC), Department of Pharmaceutical Chemistry, College of Pharmacy, King Saud University, P.O. Box 2457, Riyadh 11451, Saudi Arabia; anaglah@ksu.edu.sa (A.M.N.); mehizia@ksu.edu.sa (A.A.A.); malomar1@ksu.edu.sa (M.A.A.-O)

[2] Department of Chemistry, College of Science, Princess Nourah Bint Abdulrahman University, Riyadh 11671, Saudi Arabia; asalwasidi@pnu.edu.sa

[3] University Family Medicine Center, Department of Family and Community Medicine, College of Medicine, King Saud University Medical City, P.O. Box 2925, Riyadh 11472, Saudi Arabia; mabdallah@ksu.edu.sa

[4] Department of Biology, College of Science and Humanities, Shaqra University, Dawadmi 1101, Saudi Arabia; salsobaei@su.edu.sa

[5] Organometallic and Organometalloid Chemistry Department, National Research Centre, Dokki, Cairo 12622, Egypt; as.el-salmoon@nrc.sci.eg

[6] Biochemistry Department, Biotechnology Research Institute, National Research Centre, Dokki, Cairo 12622, Egypt; wmkamel83@hotmail.com

* Correspondence: anaglah@ksu.edu.sa (A.M.N.); as.el-salmoon@nrc.sci.eg (A.S.H.)

Table S1. Statistical correlations among the different *in vitro* biological activities of various *L. sativum* extracts at equal concentrations (100µg/mL)

		Phyto-constituents			Antioxidant		Scavenging		Anti-diabetic		Anti-Alzheimer	Anti-arthritic		Anti-inflammatory		
		**TPP**	**TCT**	**TF**	**TAC**	**IRP**	**DPPH**	**ABTS**	**α-amy**	**α-gluc**	**AChE**	**Protein Denatur.**	**Proteinase**	**COX-1**	**COX-2**	**5-LOX**
**Phyto-constituents**	**TPP**	-	0.000**	0.000**	0.000**	0.000**	0.000**	0.000**	0.000**	0.000**	0.000**	0.000**	0.000**	0.000**	0.000**	0.000**
	**TCT**	0.000**	-	0.000**	0.000**	0.000**	0.000**	0.000**	0.000**	0.000**	0.000**	0.000**	0.000**	0.000**	0.000**	0.000**
	**TF**	0.000**	0.000**	-	0.000**	0.000**	0.000**	0.000**	0.000**	0.000**	0.000**	0.000**	0.000**	0.000**	0.000**	0.000**
**Antioxidant**	**TAC**	0.000**	0.000**	0.000**	-	0.000**	0.000**	0.000**	0.000**	0.000**	0.000**	0.000**	0.000**	0.000**	0.000**	0.000**
	**IRP**	0.000**	0.000**	0.000**	0.000**	-	0.000**	0.000**	0.000**	0.000**	0.000**	0.000**	0.000**	0.000**	0.000**	0.000**
**Scavenging**	**DPPH**	0.000**	0.000**	0.000**	0.000**	0.000**	-	0.000**	0.000**	0.000**	0.000**	0.000**	0.000**	0.000**	0.000**	0.000**
	**ABTS**	0.000**	0.000**	0.000**	0.000**	0.000**	0.000**	-	0.000**	0.000**	0.000**	0.000**	0.000**	0.000**	0.000**	0.000**
**Anti-diabetic**	**α-amy**	0.000**	0.000**	0.000**	0.000**	0.000**	0.128	0.000**	-	0.000**	0.000**	0.000**	0.000**	0.000**	0.000**	0.000**
	**α-gluc**	0.000**	0.000**	0.000**	0.000**	0.000**	0.177	0.000**	0.000**	-	0.000**	0.000**	0.000**	0.000**	0.000**	0.000**
**Anti-Alzheimer**	**AChE**	0.000**	0.000**	0.000**	0.000**	0.000**	-0.363	0.000**	0.000**	0.000**	-	0.000**	0.000**	0.000**	0.000**	0.000**
**Anti-arthritic**	**Protein Denatur.**	0.000**	0.000**	0.000**	0.000**	0.000**	0.000**	0.000**	0.000**	0.000**	0.000**	-	0.000**	0.000**	0.000**	0.000**
	**Proteinase**	0.000**	0.000**	0.000**	0.000**	0.000**	0.000**	0.000**	0.000**	0.000**	0.000**	0.000**	-	0.000**	0.000**	0.000**
**Anti-inflammatory**	**COX-1**	0.000**	0.000**	0.000**	0.000**	0.000**	0.000**	0.000**	0.000**	0.000**	0.000**	0.000**	0.000**	-	0.000**	0.000**
	**COX-2**	0.000**	0.000**	0.000**	0.000**	0.000**	0.000**	0.000**	0.000**	0.000**	0.000**	0.000**	0.000**	0.000**	-	0.000**
	**5-LOX**	0.000**	0.000**	0.000**	0.000**	0.000**	0.000**	0.000**	0.000**	0.000**	0.000**	0.000**	0.000**	0.000**	0.000**	-

** indicates a positive correlation ($p \leq 0.01$).

2.1. Collection of seeds and preparation of extracts

L. sativum seeds were purchased from a local market (the Egyptian market), shade-dried, and then ground to a fine powder. Following the method demonstrated by Dixit et al. (2020), the maceration process was used to prepare the methanolic extract by dissolving 250 g of powdered seeds in 500 ml of methanol for 3 days at room temperature [33]. The Soxhlet technique was used to prepare the aqueous extract by heating the powdered seeds in the thimble with distilled water at boiling temperature. The condensation of vapors was achieved by cold running tap water dripping down into the flask through the extract. The acetone and ethyl acetate extracts were prepared by immersing 250 g of the seed powder in 1000 ml of each of the different solvents (acetone and ethyl acetate) with varying polarities, following the simple protocol proposed by George et al. (2012) [34]. Each extract was then centrifuged at 5000 rpm for 5 minutes and filtered. All filtrates were allowed to evaporate under vacuum at 40 °C until completely dry.

2.2. Phyto-chemical evaluation of L. sativum seed extracts

Total phenolic content

The concentration of total phenolic content was estimated in the different extracts using the Folin-Ciocalteu reagent with gallic acid as the standard, following the method described by Singleton and Rossi (1965) [35]. In this method, 0.1 ml of the extract was diluted to 0.5 ml with distilled water. Then, 0.25 ml of Folin-Ciocalteu reagent was added, followed by 1.25 ml of aqueous sodium carbonate solution. All tubes were vortexed and then incubated for 40 minutes at room temperature. The absorbance of the blue-colored mixtures was recorded at a wavelength of 725 nm against a blank containing 0.5 ml of distilled water instead of the extract. The concentration of total phenolic content was calculated as a gallic acid equivalent from the calibration curve of various concentrations of gallic acid standard solutions.

Total condensed tannins

Concentrations of total condensed tannins were quantified in all prepared extracts using the methods suggested by Broadhurst and Jones (1978) [36]. First, it is necessary to extract total tannins by macerating 1gm of plant tissue with acetone (70% v/v): water (tissue weight: vol., 1: 3), containing ascorbic acid (0.1% w/v). The macerate and washings were centrifuged at 2000 g for 5 min and the liquid supernatant transferred to a separating funnel. The residue was re-extracted (5x) with acetone (70% v/v). The combined extracts, on saturation with NaCl, separated into a lower aqueous and upper acetone phase. The aqueous phase was re-extracted with the upper (acetone) phase of a NaCl saturated solution of acetone (70% v/v).

The combined acetone phases were evaporated to remove acetone. Water (2 ml) was added to the solution and extracted (3x) with diethyl ether and then (3x) with ethyl acetate. The aqueous solution (containing condensed tannins) was made up to a standard volume (5 or 10ml).

The sample volume was 0.5 ml, and the total reaction volume was 5 ml. Vanillin reagent (3 ml) was added to the sample and mixed thoroughly. Concentrated hydrochloric acid (1.5 ml) was added and mixed thoroughly. The reaction mixture was allowed to stand for 15 min at 20 ± 2°C. Absorbance of the samples and blank was determined against water at 500 nm. The concentration of total condensed tannins was determined from the curve plotted between absorbance and a series of different concentrations of purified tannins (standard).

Total flavonoid content

The total flavonoid content was evaluated in the different extracts following the method reported by Arvouet-Grand *et al*. (1994) [37]. In brief, 5 mL of $AlCl_3$ (2% in methanol) was mixed with the same volume of extract solution, and the absorbance was measured at 415 nm after 10 minutes. The blank solution was prepared by mixing 5 mL of extract solution with 5 mL of methanol without the addition of $AlCl_3$. The total flavonoid content was expressed as quercetin equivalent. The standard calibration curve of quercetin was established in the range of 0-100 mg/L and the values are calculated as mg of quercetin equivalents per 100 g of extract.

2.3. In vitro studies of L. sativum seed extracts

Antioxidant activity

The total antioxidant capacity (TAC) was determined by analyzing the green phosphate/Mo^{5+} complex at a wavelength (λ) of 695 nm, following the procedure described by Prieto et al. [38]. Samples (at each concentration) were mixed with a reagent solution containing 0.3 N sulfuric acid, 28 mM sodium phosphate, and 4 mM ammonium molybdate. Methanol (80%) was used in place of the sample for the blank. The tubes were sealed and incubated in a boiling water bath for 90 minutes. After cooling to room temperature, the absorbance was measured at 695 nm against the blank. Ascorbic acid was used at the same concentrations as a standard. The antioxidant capacity was expressed as mg gallic acid equivalent per gram weight.

The iron reducing power was determined as μg/mL using the method proposed by Oyaizu (1986) [39]. In brief, 1ml of the tested sample (at each concentration) was combined with 1mL of 200mM sodium phosphate buffer (pH 6.6) and 1mL of 1% potassium ferricyanide.

The mixture was then incubated at 50°C for 20 minutes, followed by the addition of 1mL of trichloroacetic acid (10%). After centrifugation at 2000rpm for 10 minutes, the upper layer solution (2.5 mL) was mixed with 2.5 mL of double deionized water and 1mL of fresh ferric chloride (0.1%). The absorbance was measured at 700nm against a blank prepared without the sample. Ascorbic acid was used at the same concentrations as a standard. A high absorbance at 700nm indicates a higher reducing power in the reaction mixture.

Scavenging activity

The 1,1-Diphenyl-2-picryl-hydrazyl (DPPH) radical scavenging activities were evaluated using the method described by Rahman et al. [40]. An antioxidant substance capable of donating a hydrogen atom to a solution containing DPPH- can reduce the stable free radical, causing the solution to change color from violet to pale yellow. The remaining DPPH- radical was quantified by measuring the intensity of a light-purple colored DPPH methanol solution in the visible range at 518 nm using a spectroscopic method. Two milliliters of a DPPH solution (100 µM) in ethanol were mixed with 2 mL of the sample (at each concentration). The reaction mixture for each concentration was thoroughly vortexed and then incubated in the dark at room temperature for 30 minutes. The absorbance was then measured spectrophotometrically at 518 nm against a blank (ethanol). For the control, 2 mL of ethanol was added instead of the sample and run simultaneously with the test. Ascorbic acid was used at the same concentrations as a positive control. Percent inhibition of the DPPH free radical was calculated. The median inhibitory concentration (IC_{50}) for each tested compound was calculated using a series of concentrations (0, 0.75, 1.56, 3.125, 6.25, 12.5, 25, 50, and 100 µg/mL).

The procedure for the 2,2'-azinobis-(3-ethylbenzothiazoline-6-sulfonic acid) (ABTS) assay followed the method suggested by Arnao et al. [41]. Stock solutions included ABTS solution (7 mM) and potassium persulfate solution (2.4 mM). The working solution was prepared by mixing the two stock solutions in equal quantities and allowing them to react at room temperature in a dark place for 14 hours. The solution was then diluted by mixing 1 mL of ABTS solution with 60 mL of methanol to obtain an absorbance of 0.706 ± 0.01 units at 734 nm using a spectrophotometer. Fresh ABTS solution was prepared for each assay. The tested samples (at each concentration) were allowed to react with 1 mL of the ABTS solution, and the absorbance was taken at 734 nm after 7 minutes using a spectrophotometer. The ABTS scavenging capacities of the samples were compared with that of ascorbic acid (at the same concentrations) [42].

Anti-Alzheimer's activity

In this study, we assessed the inhibition percentage of the acetylcholinesterase (AChE) enzyme using Ellman's method with donepezil as the standard drug [43]. For each run, 5 µL of Acetylthiocholine (ATCh) at a concentration of 0.5 mM, 5 µL of 5,5'-dithiobis-2-nitrobenzoic acid (DTNB) at a concentration of 0.03 mM, and 5 µL of each sample (at each concentration) were added to a flat bottom 96-well plate. The mixture was then incubated for 10 minutes at 30 °C. After incubation, 5 µL of AChE at a concentration of 0.3 U/mL was added to start the reaction, and the absorbance was measured at 412nm. A control run was also performed, which included all the components except for the test sample. The median inhibitory concentration (IC_{50}) of each tested sample was calculated by plotting a curve using a series of sample concentrations against the percent of AChE inhibition.

Anti-diabetic activity

This assay involved calculating the inhibition percentage (%) of α-amylase enzyme using method based on the technique demonstrated by Wickramaratne et al. with Acarbose as the standard drug [44]. During the assay, 0.5 ml of the test solution was combined with 0.5 ml of α-amylase solution (0.5 mg/ml) and buffer (Na_2HPO_4/NaH_2PO_4 (0.02 M), NaCl (0.006 M) at pH 6.9) to create concentrations ranging from 25 to 800 µg/mL. The mixture was then left at room temperature for 10 minutes before adding 200 µL of starch solution (1% in water (w/v) buffer (Na_2HPO_4/NaH_2PO_4 (0.02 M), NaCl (0.006 M) at pH 6.9)). The reaction was stopped by adding 200 µL of DNSA (coloring) reagent (12 g of sodium potassium tartrate tetrahydrate in 8.0 mL of 2 M NaOH and 20 mL of 96 mM of DNSA solution). The test tubes were then placed in a boiling water bath (100 °C) for 10 minutes and the mixture was cooled to room temperature and diluted with 5 mL of distilled water. The absorbance was measured at 540 nm using a UV-Visible spectrophotometer. The IC_{50} of each tested sample was calculated by plotting a curve using a series of sample concentrations against the percent of α-amylase inhibition.

The inhibition percentage (%) of the α-glucosidase enzyme was determined using the method proposed by Pistia-Brueggeman and Hollingsworth with Acarbose as the standard drug [45]. Five µL of the α-glucosidase solution (10 units mL^{-1}, 0.1$moll^{-1}$ potassium phosphate buffer, pH 6.8) was pre-mixed with 10 µL of the sample solution at different concentrations (in 10% DMSO) in 620 µL of 0.1 $moll^{-1}$ potassium phosphate buffer (pH 6.8). After incubation at 37.5 °C for 20 minutes, 10 µL of p-nitro phenyl glucopyranoside (pNPG, 10 $mmoll^{-1}$) as a substrate was added to the mixture to start the reaction. The reaction mixture was then incubated at 37.5°C for 30 minutes, followed by the addition of 650 µL of 1 $moll^{-1}$ Na_2CO_3

solution to terminate the reaction. The amount of released product (p-nitro phenol) was measured at 410 nm using a UV spectrometer (UV-2550, Shimadzu, Japan) to estimate the enzymatic activity. The inhibition assay was performed in triplicate for all tests. The IC_{50} of each tested sample was calculated by plotting a curve using a series of sample concentrations against the percent of α-glucosidase inhibition.

Anti-arthritic activity

In the anti-arthritic activity study, this assay involved determining the percentage of protein denaturation [46] and proteinase [47] inhibition using diclofenac sodium as the standard non-steroidal anti-inflammatory drug, as prepared according to Meera et al. [48]. The protein denaturation percentage was measured by mixing 0.5mL of the test control solution, prepared by combining 0.45 mL of bovine serum albumin (BSA) (5% w/v aqueous solution) with 0.05 mL of distilled water. Then, 0.05 mL of the test solution was added to 0.45 mL of distilled water to form the product control (0.5 mL). The different samples (test solution) and diclofenac sodium (standard) were used. The pH value in all prepared solutions was adjusted to 6.3 using HCl (1N). All the samples were incubated at 37 ºC for 20 min, and the temperature was then increased to 57 ºC, maintaining the samples at that degree for 3 min. After cooling, 2.5 mL of phosphate buffer was added to the prepared solutions. The absorbance was determined at 416 nm using a UV-Visible spectrophotometer. The percentage of protein denaturation inhibition can be calculated. Proteinase inhibitory activity was assessed by combining the test sample (1 mL) with a reaction mixture containing 0.06 mg trypsin dissolved in 1 mL of 20 mM Tris HCl buffer (pH 7.4). The mixture was then incubated for 5 minutes at 37°C, followed by the addition of 1 mL of casein (0.8% w/v). After an additional 20 minutes of incubation, 2 mL of perchloric acid (70%) was added to stop the reaction. The cloudy suspension was then centrifuged, and the absorbance of the supernatant was measured at 210 nm against buffer as the blank. The percentage of proteinase inhibitory activity was then calculated.

Anti-inflammatory activity

In vitro anti-inflammatory evaluation was performed through inhibition of two isoenzymes cyclooxygenase COX-1 and COX-2 (ovine/human) [49], along with 5-LOX enzyme (human recombinant). COX‑1 and COX‑2 inhibition assay has been performed by means of COX-1 and COX-2 kit (Cayman, No.: 560131), where different known concentrations of the tested compounds were added separately to a mixture of 10 µL of COX-1 or COX-2 and 0.1 M HCl buffer, left for incubation at room temperature for 10 min. After that, 10µL of arachidonic

acid, fifty µL HCl and Ellman's reagent have been added. The absorbance has been determined at UV-410 nm alongside blank; IC_{50} has been determined via linear regression.

5‑Lipoxygenase inhibition assay [50] was carried out by 5-LOX kit (No. 437996, Sigma-Aldrich), where different concentrations of the tested compounds were added to 90 µL from 5-LOX, 100 µL of de chromogen, then 10 µL from arachidonic acid was added and shaken for 10 min, the absorbance has been determined at UV-490 nm compared to blank. IC50 has been calculated through linear regression.

Cytotoxic activity and Enzymatic Assay

It will be assayed against human hepatocellular carcinoma (HepG-2), colon cancer (Caco-2), and lung cancer (A549) cells using 3-(4,5-dimethythiazol-2-yl)-2,5-diphenyl tetrazolium bromide (MTT) assay by determining the optical density (OD) at 570 nm according to the method suggested by Vichai and Kirtikara [51]. The cells were dispensed in a 96-well sterile microplate (3×10^4 cells/ well), followed by their incubation at 37 °C with a series of different concentrations of 10 µL of each compound or doxorubicin (positive control, in DMSO) for 48 h in a serum free medium prior to the MTT assay. Subsequently, the media were carefully removed, and 40 µL of MTT (2.5 mg/mL) was added to each well and then incubated for an additional 4 h. Purple formazan dye crystals were solubilized by the addition of 200 µL of DMSO. The absorbance was measured at 570 nm using a SpectraMax Paradigm Multi-Mode microplate reader. The relative cell viability was expressed as the mean percentage of viable cells relative to the untreated control cells. All experiments were conducted in triplicate. Percent of the cell-growth inhibition (%) and the IC_{50} was calculated using IC_{50} calculation software.

Activities of caspase-3 were measured by enzyme-linked immunosorbent assay (ELISA) using the Invitrogen caspase-3 (Active) (human) ELISA kit (96 tests) from Invitrogen Corporation, following the manufacturer's instructions. Activities of Bcl-2 were measured using the Invitrogen Zymed Bcl-2 ELISA Kit (96 tests) from Invitrogen Corporation, following the manufacturer's instructions [52-54].

2.4. In vivo studies of L. sativum seed extracts

2.4.1. Median lethal dose (LD_{50})

The LD_{50} of the different studied extracts were studied separately. One hundred eighty six (186) adult albino mice weighing 20-25 g were divided into 31 groups, with 6 mice in each group. These groups were used to calculate the LD_{50} of the methanolic, aqueous, acetone and ethyl acetate extracts. For each extract, the groups were orally treated with increasing doses (500, 1000, 2000, 4000, 6000, 8000, 10000, 12000, and 14000 mg/Kg) using a stomach tube. The LD_{50} was determined by counting the number of dead mice after 24 hours of oral administration. The LD_{50} was calculated using the equation suggested by Paget and Barnes (1964) [55].

Table S2: Data used for calculating the median lethal dose (LD_{50}) of methanolic *L. sativum* seed extract in mice.

Dose (mg/kg)	No of live animals/group	No of dead animals	Z	d	Z×d
**1000**	6	0	0	1000	0
**2000**	6	0	0.5	2000	1000
**4000**	5	1	1	2000	2000
**6000**	5	1	1.5	2000	3000
**8000**	4	2	2.5	2000	5000
**10000**	3	3	4	2000	8000
**12000**	1	5	5.5	2000	11000
**14000**	0	6	3	0	0

$$LD_{50} = Dm - \{\Sigma(Z \times d) / n\}$$

Where:

Dm: The dose by which all the mice died.

Z: Half the sum of dead mice from two successive doses.

d: The difference between the two successive doses.

N: Number of mice in each group.

LD_{50} = 14000 – (30000/6)

LD_{50} = **9000 mg/kg**.

The therapeutic doses:

- LD_{50}/10 = **900 mg/kg**.
- LD_{50}/20 = **450 mg/kg**.

Table S3: Data used for calculating the median lethal dose (LD_{50}) of aqueous *L. sativum* seed extract in mice.

**Dose** (mg/kg)	**No of live animals/group**	**No of dead animals**	**Z**	**d**	**Z×d**
**1000**	6	0	0	1000	0
**2000**	6	0	0.5	2000	1000
**4000**	5	1	1	2000	2000
**6000**	5	1	1.5	2000	3000
**8000**	4	2	3.5	2000	7000
**10000**	1	5	5	2000	10000
**12000**	1	5	5.5	2000	11000
**14000**	0	6	3	0	0

$$\mathbf{LD_{50} = Dm - \{\Sigma(Z\times d) / n\}}$$

<u>Where:</u>

Dm: The dose by which all the mice died.

Z: Half the sum of dead mice from two successive doses.

d: The difference between the two successive doses.

N: Number of mice in each group.

LD_{50} = 14000 – (34000/6)

LD_{50} = **8333.33 mg/kg**.

The therapeutic doses:

- LD_{50}/10 = **833.33 mg/kg**.
- LD_{50}/20 = **416.67 mg/kg**.

Table S4: Data used for calculating the median lethal dose (LD_{50}) of acetone *L. sativum* seed extract in mice.

Dose (mg/kg)	No of live animals/group	No of dead animals	Z	d	Z×d
**500**	6	0	0	500	0
**1000**	6	0	0	1000	0
**2000**	6	0	1	2000	2000
**4000**	4	2	2	2000	4000
**6000**	4	2	2.5	2000	5000
**8000**	3	3	4	2000	8000
**10000**	1	5	5.5	2000	11000
**12000**	0	6	3	0	0

$$LD_{50} = Dm - \{\Sigma(Z \times d) / n\}$$

Where:

Dm: The dose by which all the mice died.

Z: Half the sum of dead mice from two successive doses.

d: The difference between the two successive doses.

N: Number of mice in each group.

LD_{50} = 12000 – (30000/6)

LD_{50} = **7000 mg/kg**.

The therapeutic doses:

- LD_{50}/10 = **700 mg/kg**.
- LD_{50}/20 = **350 mg/kg**.

Table S5: Data used for calculating the median lethal dose (LD_{50}) of ethyl acetate *L. sativum* seed extract in mice; biological methods, and statistical analysis.

Dose (mg/kg)	No of live animals/group	No of dead animals	Z	d	Z×d
**500**	6	0	0.5	500	250
**1000**	5	1	1	1000	1000
**2000**	5	1	1.5	2000	3000
**4000**	4	2	2	2000	4000
**6000**	4	2	3.5	2000	7000
**8000**	1	5	5.5	2000	11000
**10000**	0	6	3	0	0

$$LD_{50} = Dm - \{\Sigma(Z \times d) / n\}$$

Where:
Dm: The dose by which all the mice died.
Z: Half the sum of dead mice from two successive doses.
d: The difference between the two successive doses.
N: Number of mice in each group.

LD_{50} = 10000 – (26250/6)
LD_{50} = **5625 mg/kg**
The therapeutic doses:
- LD_{50}/10 = **562.50 mg/kg**.
- LD_{50}/20 = **281.25 mg/kg**.

2.4.2. Experimental Design

A total of forty-five (45) healthy adult male mice, weighing between 20 and 30 g, were kept in the Animal House at the National Research Centre in Dokki, Cairo, Egypt. The mice were maintained under controlled environmental and nutritional conditions and had unlimited access to water. They were housed in nine cages, with five mice per cage, and were randomly divided as follows:

Control group: Mice were fed with a normal diet and received tap water.

Methanolic *L. sativum* seed extract treated groups: mice were subdivided into 2 groups. One group was treated with methanolic extract at a dose of 900 mg/kg (1/10 of LD_{50}) orally and the other group was treated with the extract at a dose of 450 mg/kg (1/20 of LD_{50}) for 30 days.

Aqueous *L. sativum* seed extract treated groups: mice were subdivided into 2 groups. One group was treated with aqueous extract at a dose of 833.33 mg/kg orally and the other group was treated with the extract at a dose of 416.67 mg/kg for 30 days.

Acetone *L. sativum* seed extract treated groups: mice were subdivided into 2 groups. One group was treated with aqueous extract at a dose of 700 mg/kg and the other group was treated with the extract at a dose of 350 mg/kg for 30 days.

Ethyl acetate *L. sativum* seed extract treated groups: mice were subdivided into 2 groups. One group was treated with aqueous extract at a dose of 562.50 mg/kg and the other group was treated with the extract at a dose of 281.25 mg/kg for 30 days.

2.4.3. Samples collection

The last treatment dose was followed by an 18-hour fasting period before anesthesia. For hematological measurements, blood samples were collected from the retro-orbital plexus using heparinized tubes and the capillary puncture method described by Sorg and Buckner (1964) [56]. Other blood samples were centrifuged at 3000 rpm for 15 minutes to allow clotting, and the separated sera were stored at -20 °C until the biochemical assay. Liver, kidney and spleen tissues were obtained from sacrificed mice and dissected into two parts. Part I was immediately preserved in glutaraldehyde (10%) for microscopic examination. Part II was homogenized in Tris-HCl buffer (0.01 M, pH 7.4), centrifuged at 10,000 rpm for 15 minutes, and the clear supernatants were transferred to new tubes and stored at -20 °C for biochemical assays.

2.4.4. Biochemical assays

2.4.4.1. Hematological and biochemical measurements

The hematological measurements, including red blood cells (RBCs), hemoglobin (HB), hematocrit (HCT), platelet count (PLT), and white blood cells (WBCs), were determined using an automatic blood analyzer called ABX Micros 60, manufactured by HORIBA ABX SAS. These measurements were taken from heparinized blood samples. The commercially available kits (Spectrum Diagnostics Egyptian Company for Biotechnology in Cairo, Egypt) were used for determining all conventional biochemical measurements (liver (serum alanine transaminase (ALT), aspartate transaminase (AST), alkaline phosphatase (ALP) and gamma-glutamyl transferase (GGT), kidney (urea, creatinin, blood urea nitrogen (BUN), total protein and albumin), heart functions (creatin kinase (CK) and lactate dehydrogenase (LDH)), as well as lipid profiles (total cholesterol (TC), triglycerides (TG), and high-density lipoprotein-cholesterol (HDL-c)) in the serum specimens. The low-density lipoprotein-cholesterol (LDL-c) was calculated using the formula proposed by Schumann and Klauke (2003) [57].

2.4.4.2. Biochemical assays in supernatants of tissues homogenates

Oxidative stress markers [58-64], such as total antioxidant capacity (TAC) and reduced glutathione (GSH), along with the activities of superoxide dismutase (SOD), catalase (CAT), and glutathione peroxidase (GPx) enzyme, were assessed in liver and brain tissue homogenates. Furthermore, the products of lipid peroxidation (LPO) as well as the total protein carbonyl (TPC) concentration were also quantified [65, 66].

2.5. Statistical analysis

Statistical analysis was performed using the Statistical Package for the Social Sciences (SPSS) for Windows, version 11.0. One-way analysis of variance (ANOVA) was utilized to evaluate both positive and negative correlations between in vitro biological activities. The significance threshold for identifying significant associations was a "*p*" value less than 0.05.

References

[34] George, V.C.; Kumar, D.R.; Rajkumar, V.; Suresh, P.K.; Kumar, R.A. Quantitative assessment of the relative antineoplastic potential of the n-butanolic leaf extract of *Annona muricata Linn.* in normal and immortalized human cell lines. *Asian Pac. J. Cancer Prev.* **2012**, *13*, 699-704. https://doi.org/10.7314/apjcp.2012.13.2.699

[35] Singleton, V.L.; Rossi, J.A. Colorimetry of total phenolics with phosphomolybdicphosphotungstic acid reagents. *Am. J. Enol. Vitic.* **1965**, *16*, 144-158. (doi: 10.5344/ajev.1965.16.3.144).

[36] Broadhurst, R.B.; Jones, W.T. Analysis of condensed tannins using acidified vanillin. *J. Sci. Food Agric*. **1978**, *29*, 788-794. https://doi.org/10.1002/jsfa.2740290908

[37] Arvouet-Grand, A.; Vennat, B.; Pourrat, A.; Legret, P. Standardization of propolis extract and identification of principal constituents. *J. Pharm. Belg.* **1994**, *49*, 462-468.

[38] Prieto, P.; Pineda, M.; Aguilar, M. Spectrophotometric quantitation of antioxidant capacity through the formation of a phosphomolybdenum complex: Specific application to the determination of vitamin E. *Anal. Biochem*. **1999**, *269*, 337-341. https://doi.org/10.1006/abio.1999.4019 (doi: 10.1006/abio.1999.4019).

[39] Oyaizu, M. Studies on product of browning reaction prepared from glucose amine. *Jpn. J. Nutr. Diet.* **1986**, *44*, 307–315. https://doi.org/10.5264/eiyogakuzashi.44.307

[40] Rahman, M.M.; Islam, M.B.; Biswas, M.; Alam, A.K. In vitro antioxidant and free radical scavenging activity of different parts of *Tabebuia pallida* growing in Bangladesh. *BMC Res. Notes* **2015**, *8*, 621. https://doi.org/10.1186/s13104-015-1618-6

[41] Arnao, M.B.; Cano, A.; Acosta, M. The hydrophilic and lipophilic contribution to total antioxidant activity. *Food Chem.* **2001**, *73*, 239–244. https://doi.org/10.1016/S0308-8146(00)00324-1

[42] Chakraborthy, G.S. Free radical scavenging activity of *Costus speciosus* leaves. *Indian J. Pharm. Educ. Res.* **2009**, *43*, 96–98.

[43] Ellman, G.L.; Courtney, K.D.; Andres, V.J.; Featherstone, R.M. A new and rapid colorimetric determination of acetylcholinesterase activity. *Biochem. Pharmacol.* **1961**, *7*, 88–95. https://doi.org/10.1016/0006-2952(61)90145-9

[44] Wickramaratne, M.N.; Punchihewa, J.; Wickramaratne, D. In-vitro alpha amylase inhibitory activity of the leaf extracts of *Adenanthera pavonina*. *BMC Complement. Altern. Med.* **2016**, *16*, 466. https://doi.org/10.1186/s12906-016-1452-y

[45] Pistia-Brueggeman, G.; Hollingsworth, R.I. A preparation and screening strategy for glycosidase inhibitors. *Tetrahedron* **2001**, *57*, 8773–8778. https://doi.org/10.1016/S0040-4020(01)00877-8

[46] Das, S.; Sureshkumar, P. Effect of methanolic root extract of *Blepharispermum subsesssile* DC in controlling arthritic activity. *Res. J. Biotechnol*. **2016**, *11*, 65–74.

[47] Oyedapo, O.O.; Famurewa, A.J. Antiprotease and Membrane Stabilizing Activities of Extracts of *Fagara Zanthoxyloides*, *Olax Subscorpioides* and *Tetrapleura Tetraptera*. *Int. J. Pharmacogn*. **1995**, *33*, 65–69. https://doi.org/10.3109/13880209509088150

[48] Meera, S.; Ramaiah, N.; Kalidindi, N. Illustration of anti-rheumatic mechanism of rheumavedic capsule. *Saudi Pharm. J.* **2011**, *19*, 279–284. https://doi.org/10.1016/j.jsps.2011.07.002

[49] Alaa, A.M.; El-Azab, A.S.; Abou-Zeid, L.A.; ElTahir, K.E.; Abdel-Aziz, N.I.; Ayyad, R.R.; Al-Obaid, A.M. Synthesis, anti-inflammatory, analgesic and COX-1/2 inhibition activities of anilides based on 5, 5-diphenylimidazolidine-2, 4-dione scaffold: molecular docking studies. *Eur. J. Med. Chem*. **2016**, *115*, 121-131. https://doi.org/10.1016/j.ejmech.2016.03.011

[50] Huang, Y.; Zhang, B.; Li, J.; Liu, H.; Zhang, Y.; Yang, Z.; Liu, W. Design, synthesis, biological evaluation and docking study of novel indole-2-amide as anti-inflammatory agents with dual inhibition of COX and 5-LOX. *Eur. J. Med. Chem.* **2019**, *180*, 41-50. https://doi.org/10.1016/j.ejmech.2019.07.004

[51] Vichai, V.; Kirtikara, K. Sulforhodamine B colorimetric assay for cytotoxicity screening. *Nat. Protoc.* **2006**, *1*, 1112–1116. https://doi.org/10.1038/nprot.2006.179

[52] Hassan, A.S., Mady, M.F., Awad, H.M.; Hafez, T.S. Synthesis and antitumor activity of some new pyrazolo[1, 5-a] pyrimidines. *Chin. Chem. Lett.* **2017**, *28,* 388-393.

[53] Hassan, A.S.; Awad, H.M.; Magd-El-Din, A.A.; Hafez, T.S. Synthesis and in vitro antitumor evaluation of novel Schiff bases. *Med. Chem. Res.* **2018**, *27*, 915–927. https://doi.org/10.1007/s00044-017-2113-5

[54] Pandey, P.; Khan, F.; Alzahrani, F.A.; Qari, H.A.; Oves, M. A Novel Approach to Unraveling the Apoptotic Potential of Rutin (Bioflavonoid) via Targeting Jab1 in Cervical Cancer Cells. *Molecules* **2021**, *26*, 5529. https://doi.org/10.3390/molecules26185529

[55] Paget, G.E.; Barnes, J.M. Chapter 6- Toxicity tests. In: *Evaluation of Drug Activities: Pharmacometrics*; Laurance, D.R.; Bacharach, A.L., Eds.; Academic Press, 1964, Vol 1, 135-166. https://doi.org/10.1016/B978-1-4832-2845-7.50012-8

[56] Sorg, D.A.; Buckner, B. A simple method of obtaining venous blood from small laboratory animals. *Proc. Soc. Exp. Biol. Med.* **1964**, *115*, 1131-1132. https://doi.org/10.3181/00379727-115-29134

[57] Schumann, G.; Klauke, R. New IFCC reference procedures for the determination of catalytic activity concentrations of five enzymes in serum: preliminary upper reference limits obtained in hospitalized subjects. *Clin. Chim. Acta* **2003**, *327*, 69-79. https://doi.org/10.1016/s0009-8981(02)00341-8

[58] Koracevic, D.; Koracevic, G.; Djordjevic, V.; Andrejevic, S.; Cosic, V. Method for the measurement of antioxidant activity in human fluids. *J. Clin. Pathol.* **2001**, *54*, 356-361. https://doi.org/10.1136/jcp.54.5.356

[59] Beutler, E.; Duron, O.; Kelly, B.M. Improved method for the determination of blood glutathione. *J. Lab. Clin. Med.* **1963**, *61*, 882-888.

[60] Nishikimi, M.; Appaji, N.; Yagi, K. The occurrence of superoxide anion in the reaction of reduced phenazine methosulphate and molecular oxygen. *Biochem. Biophys. Res. Comm*. **1972**, *46*, 849-864. https://doi.org/10.1016/S0006-291X(72)80218-3

[61] Aebi, H. Catalase in vitro. *Methods Enzymol.* **1984**, *105*, 121-126. https://doi.org/10.1016/S0076-6879(84)05016-3

[62] Paglia, D.E.; Valentine, W.N. Studies on the Quantitive and Qualitative Charecterization of Erythrocyte Glutathione Peroxidase. *J. Lab. Clin. Med.* **1967**, *70*, 158-163.

[63] Ohkawa, H.; Ohishi, N.; Yagi, K. Assay for lipid peroxides in animal tissues by thiobarbituric acid reaction. *Anal. Biochem.* **1979**, *95*, 351-358. https://doi.org/10.1016/0003-2697(79)90738-3

[64] Levine, R.L.; Williams, J.A.; Stadtman, E.R.; Shacter, E. Carbonyl assays for determination of oxidatively modified proteins. *Methods Enzymol.* **1994**, *233*, 346-357. https://doi.org/10.1016/s0076-6879(94)33040-9

[65] Engelmann, H.; Novick, D.; Wallach, D. Two tumor necrosis factor-binding proteins purified from human urine. Evidence for immunological cross-reactivity with cell surface tumor necrosis factor receptors. *J. Biol. Chem.* **1990**, *265*, 1531-1536. https://doi.org/10.1016/S0021-9258(19)40049-5

[66] March, C.J.; Mosley, B.; Larsen, A.; Cerretti, D.P.; Braedt, G.; Price, V.; Gillis, S.; Henney, C.S.; Kronheim, S.R.; Grabstein, K.; Conlon, P.J.; Hopp, T.P.; Cosman, D. Cloning, sequence and expression of two distinct human interleukin-1 complementary DNAs. *Nature* **1985**, *315*, 641-647. https://doi.org/10.1038/315641a0
